# On-Chip Micro Temperature Controllers Based on Freestanding Thermoelectric Nano Films for Low-Power Electronics

**DOI:** 10.1007/s40820-024-01342-3

**Published:** 2024-02-20

**Authors:** Qun Jin, Tianxiao Guo, Nicolás Pérez, Nianjun Yang, Xin Jiang, Kornelius Nielsch, Heiko Reith

**Affiliations:** 1 Institute for Metallic Materials, Leibniz Institute for Solid State and Materials Research, 01069 Dresden, Germany; 2 Institute of Materials Engineering, University of Siegen, 57076 Siegen, Germany; 3 Institute of Applied Physics, Technical University of Dresden, 01069 Dresden, Germany; 4 Institute of Materials Science, Technical University of Dresden, 01069 Dresden, Germany

**Keywords:** Temperature control, Low-power electronics, On-chip micro temperature controller, Freestanding thermoelectric nano films, Temperature-sensitive components

## Abstract

Multidimensional integration and multifunctional component assembly have been greatly explored in recent years to extend Moore’s Law of modern microelectronics. However, this inevitably exacerbates the inhomogeneity of temperature distribution in microsystems, making precise temperature control for electronic components extremely challenging. Herein, we report an on-chip micro temperature controller including a pair of thermoelectric legs with a total area of 50 × 50 μm^2^, which are fabricated from dense and flat freestanding Bi_2_Te_3_-based thermoelectric nano films deposited on a newly developed nano graphene oxide membrane substrate. Its tunable equivalent thermal resistance is controlled by electrical currents to achieve energy-efficient temperature control for low-power electronics. A large cooling temperature difference of 44.5 K at 380 K is achieved with a power consumption of only 445 μW, resulting in an ultrahigh temperature control capability over 100 K mW^−1^. Moreover, an ultra-fast cooling rate exceeding 2000 K s^−1^ and excellent reliability of up to 1 million cycles are observed. Our proposed on-chip temperature controller is expected to enable further miniaturization and multifunctional integration on a single chip for microelectronics.

**Supplementary Information:**

The online version contains supplementary material available at 10.1007/s40820-024-01342-3.

## Highlights

- Dense and flat freestanding Bi_2_Te_3_-based thermoelectric nano films were successfully fabricated by sputtering technology using a newly developed nano graphene oxide membrane as a substrate.
- On-chip micro temperature controllers were integrated using conventional micro-electromechanical system technology, to achieve energy-efficient temperature control for low-power electronics.
- The tunable equivalent thermal resistance enables an ultrahigh temperature control capability of 100 K mW^−1^ and an ultra-fast cooling rate exceeding 2000 K s^−1^, as well as excellent reliability of up to 1 million cycles.

**Supplementary Information:**

The online version contains supplementary material available at 10.1007/s40820-024-01342-3.

## Graphical abstract

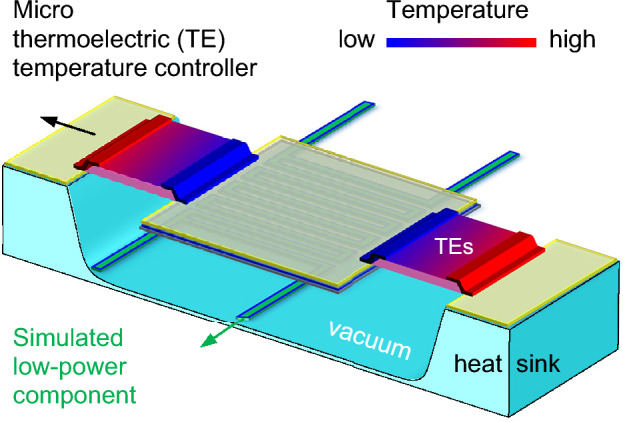

## Introduction

Temperature control is critical in modern electronics, due to the multiple effects of temperature on the performance of almost all microelectronic devices [1–3], as diverse as accuracy [4], sensitivity [5, 6], reliability [7, 8], stability [9, 10] and adjustability [11–14] of electronic components. Typically, temperature changes mainly come from diurnal and seasonal temperature variations of the external environment [15] (even a fluctuation of ~ 40 K/day or ~ 80 K/year) and the inevitable heating effect caused by operating internal high-power components (e.g., near-field wireless transmission of data [16, 17] and power [18, 19]) in the same microsystems. Moreover, the miniaturization of electronics towards high-performance and low-power consumption [20] and the diversification of demand for multidimensional integrated devices [21] in the Internet of Things make the temperature distribution of multifunctional microsystems manifest with spatial inhomogeneity and temporal uncertainty [22], leading to further challenges in thermal management [23, 24].

**Fig. 1:**
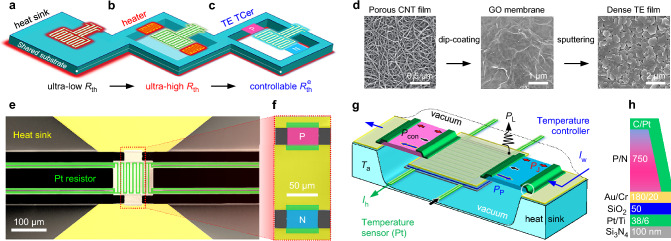
Design concept of the on-chip micro temperature controllers. Schematics of temperature control through **a** cooling by a heat sink with an ultra-low intrinsic thermal resistance $R_{\text{th}}$ for high-power electronics, **b** heating by a microheater with an ultra-high $R_{\text{th}}$ and **c** controlling by a micro thermoelectric (TE) temperature controller with a widely controllable equivalent thermal resistance ${R}_{\text{th}}^{\text{ e}}$ for low-power electronics. Note that the heating effect in **a** mainly causes the temperature fluctuations in **b** and **c** through their shared substrate. **d** SEM images of the nanoporous carbon nanotube (CNT) film, graphene oxide (GO) nano membrane by dip-coating and dense TE film by sputtering, respectively. **e** Top view false-colour SEM image of micro temperature controller (μ-TCer), showing the lateral multilevel microstructures. **f** Close-up image of TE legs marked in **e**. **g** 3D schematic of the TCer in vacuum, composed of a TE temperature control circuit driven by working current *I*_w_ (blue arrow), and a Pt sensor to measure temperature and simulate low-power electronics using a heating current *I*_h_ (green arrow). **h** Schematic vertical multilayer nanostructures of the μ-TCer marked with a white circle in **g**

Heat transfer engineering is particularly important for temperature control, including passive heat transfer generally induced by a temperature difference in space, and active heat transfer driven by external physical fields [25]. To enhance solid-state thermal conduction, reducing thermal resistance [26, 27] between high-power electronics and their environment is the most widely studied passive heat dissipation strategy (demonstrated in Fig. 1a). Heat conduction can be significantly improved when combined with thermal convection using commercial fan cooling at the system level or liquid cooling at the chip level [28]. In general, two drawbacks of the above overall heat dissipation methods exist: they are not suitable for achieving local temperature stabilization at the component level; and they are limited to passively lowering the temperature difference (Δ*T* = *T*_s_ − *T*_a_) between the power electronics (set temperature *T*_s_) and their environment (ambient temperature *T*_a_). Paradoxically, lowering Δ*T*, in return, reduces their heat dissipation power (proportional to Δ*T*), resulting in poor temperature control.

Microheaters based on the Joule effect can maximize the performance of temperature-sensitive components by actively providing a stable set temperature at the microscale [10–14], which can enable a micro-electromechanical system (MEMS) resonator to achieve ppb frequency stability over external temperature variations of up to 100 K [10], and the temperature control capability can exceed 5 K mW^−1^ after increasing the thermal resistance *R*_th_ (as shown in Fig. 1b). While this overheating method results in increased system power consumption and heat dissipation, and inevitably leads to performance degradation and shortened lifetime of electronics, this is the only on-chip temperature stabiliser available to date. Therefore, a micro temperature controller (μ-TCer) with the ability to actively control Δ*T* by adjusting the equivalent thermal resistance (${R}_{\text{th}}^{\text{ e}}$ = Δ*T*/*P*_h_) for the low-power component (with heating power *P*_h_) is highly advantageous. While intrinsic thermal resistance *R*_th_ is determined solely by material properties and geometry, ${R}_{\text{th}}^{\text{ e}}$ can be controlled by the ambient conditions (convection and radiation), active and passive heat transport in the system. In particular, ${R}_{\text{th}}^{\text{ e}}$ can be widely tuned by active solid-state cooling technologies based on the caloric [29] and thermoelectric [30] effects. A comprehensive comparison with the caloric coolers shows that an electronically controllable thermoelectric cooler (TEC) is more favourable owing to its stable device structure, and the advantages of not causing noise, vibration or volume changes in service. Particularly, thin-film micro TECs compatible with the integrated electronics exhibit high cooling power density and ultra-fast response [31–34].

The in-plane freestanding TEC based on nanograined SiGe films can achieve a cooling temperature of 10.3 K, but the low figure of merit of SiGe films (~ 0.14) needs to be improved [35]. A cooling temperature of up to 14 K was achieved recently by integrating high-performance Bi_2_Te_3_-based films with ordered microstructures into micro TECs, however, interface problems still hinder further improvement [36]. Therefore, the integration of high-performance TE nano films prepared by bottom-up strategies [37] into a micro TCer to achieve energy-efficient and wide-range temperature control for low-power components remains a major challenge. In this work, we present an on-chip μ-TCer based on a co-design concept that combines a high intrinsic *R*_th_ and a widely controllable ${R}_{\text{th}}^{\text{ e}}$ (schematically shown in Fig. 1c), enabling efficient and wide-range temperature control for low-power microelectronics. We have obtained dense and flat freestanding Bi_2_Te_3_-based TE nano films by sputtering technology using a newly developed ultra-thin graphene oxide membrane as a substrate (Fig. 1d), which were integrated into the μ-TCers using conventional MEMS technology (see Fig. S1 for detailed integration process). Figure 1e–h shows the structure of the μ-TCer, mainly consisting of a unicouple of 750-nm-thick n- and p-type TE legs with a length of ~ 25 μm and a width of ~ 50 μm, detailed parameters are summarized in Tables S1and S2. This optimized μ-TCer exhibits a remarkable temperature control capability of 100 K mW^−1^ and a fast response time of 5 ms, as well as excellent operational stability and cycle durability.

## Experimental Section

### Fabrication of Freestanding TE Nano Films

The carbon nanotube films prepared by vacuum filtration of carbon nanotube solution were first transferred onto SiN/Si frames (Fig. S2a−c). Then monolayer graphene oxide nanosheets were covered on carbon nanotube films by dip-coating method to form ultra-thin hybrid nano membranes (Fig. S2d, e), details in Sect. S1.1. High-purity Sb_2_Te_3_ (99.99%), Bi_2_Te_3_ (99.99%) and Bi_2_Se_3_ (99.99%) targets were used for co-sputtering p-type (Bi-doped Sb_2_Te_3_) and n-type (Se-doped Bi_2_Te_3_) films on above nanoporous carbon nanotube film and nanometre-thick hybrid membrane substrates, with a base chamber pressure of ~ 5.0 × 10^−4^ mTorr and Ar operating pressure of ~ 5 mTorr at 580 K with a ~ 30 min annealing before device integration and performance characterization. Controlling the deposition rate of the individual targets by varying the sputtering power can precisely regulate the chemical composition of the TE films.

### Materials and Performance Characterization

The microstructures and phase purity of the samples were characterized by X-ray diffraction (XRD, 3000 PTS diffractometer, GE Inspection Technologies GmbH, Cu radiation) and scanning electron microscopy (SEM, Zeiss ultra55, Germany), and Energy-dispersive spectra (EDS) were used to characterize the compositions of the samples. The thicknesses of the TE films were measured by cross-sectional SEM images (Fig. S2). The standard four-probe method (LSR-3, Linseis) was used to simultaneously measure the in-plane temperature-dependent Seebeck coefficient (*α*) and electrical conductivity (*σ*) with the He gas protection from room temperature to 420 K. The power factor (*PF*) was calculated according to the relation *PF* = *α*^2^ × *σ*. The measurement uncertainties for *σ*, *α* and *PF* were less than 2%, 5%, and 12%, respectively. The Hall electrical conductivity was measured with a physical property measurement system (PPMS) system based on the van der Pauw method, used for electrical conductivity calibration.

### Integration and Measurement of Micro TCers

The fabrication process of the on-chip TE TCers is shown in Fig. S1. Beginning with a 1 × 1 cm^2^ silicon/silicon nitride (Si/Si_3_N_4_) substrate, including ~ 300-μm-thick Si and ~ 100-nm-thick double-sided Si_3_N_4_ layers, the Si_3_N_4_ layer was partially etched by reactive ion etching (RIE) using CF_4_ gas under the protection of the patterned photoresist. The freestanding Si_3_N_4_ window was obtained after removing the Si layer in the 40% KOH at 353 K for ~ 300 min. Second, temperature sensor electrodes (Pt/Ti, 38/6 nm, marked by green in Fig. 1e–h) were deposited on the Si_3_N_4_ window using standard lithography, magnetron sputtering, and lift-off process, followed by a 50-nm-thick Atomic Layer Deposition (ALD) of SiO_2_ at 473 K as an insulation layer. Next, the photoresist was lithographically patterned on the SiO_2_ layer, to open the electrodes for performance measurement by RIE with CF_4_ gas. Third, working electrodes (Au/Cr, 180/20 nm, marked by yellow in Fig. 1e–h) can be obtained by sputtering and patterned etching. It is worth noting that the key role of the above SiO_2_ layer is to insulate the Pt sensor and Au working electrodes in TCer (illustrated in the multilayer structure in Fig. 1g). Fourth, the unnecessary parts of the Si_3_N_4_ window together with SiO_2_ are thoroughly etched by RIE with CF_4_ gas to ensure good thermal insulation conditions for the μ-TCer. Finally, the individually prepared n- and p-type freestanding Bi_2_Te_3_-based TE thin films (Sect. 2.1) were integrated onto the above-prepared chips by the focused dual-beam technique (FEI, Helios 600i). The integrated on-chip μ-TCer is shown in the SEM image (Fig. 1e, f) and the corresponding 3D schematic (Fig. 1g, h), details in Tables S1 and S2. The PPMS system can provide a high vacuum (~ 0.01 mTorr) and accurate ambient temperature setting (280−380 K) for the performance test of our μ-TCers (similar to the operating temperature range of microelectronics). A Pt temperature sensor was used to assess the temperature controllability of our TCers (Fig. S3). Heat-compensation method (dual currents) was used for the cooling power and efficiency test, one is the working current (*I*_w_), and another one is the heating current (*I*_h_) to simulate the Joule effect of the micro components and simultaneously monitor the real-time temperature, details in Sect. S1.2.

### Heat Transfer in Power Electronics

**Fig. 2:**
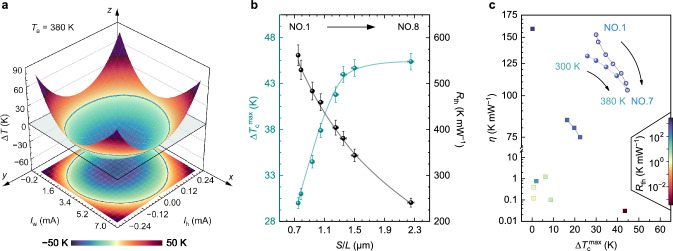
Cooling performance characterization. **a** Dependence of temperature difference Δ*T* on working current *I*_w_ and heating current *I*_h_ of the μ-TCer with optimized geometric parameters. **b** $\Delta T_{{\text{c}}}^{ \max }$ (green) and $R_{\text{th}}$ (black) as a function of the section-to-length ratio (*S*/*L*) in a series of μ-TCers (NO.1–NO.8) and their fitting lines. **c** Comparisons of cooling performance of the previously reported micro TECs (square) and μ-TCers in this work (circle), including ${\Delta T}_{{\text{c}}}^{{\text{ max}}}$ (*P*_c_ = 0 W) and corresponding cooling temperature control capability *η*. Note that the colour bar indicates their intrinsic $R_{\text{th}}$ valves, detailed data and related references are provided in Tables S1 and S3

To stabilize the temperature of a low-power temperature-sensitive component in a closed system, its heating power *P*_h_ will be completely transferred to the heat sink by net cooling power *P*_c_ (*P*_h_ = *P*_c_), and *P*_c_ can be described by the heat transfer equation [38]:

1 $$P_{{\text{c}}} = P_{{\text{P}}} - P_{{\text{J}}} /2 - P_{{{\text{con}}}} - P_{{\text{L}}}$$

where *P*_P_, *P*_J_, *P*_con_ and *P*_L_ are the Peltier cooling and Joule heating power, conductive heat power, and convective and radiative heat loss power, respectively. Since *P*_h_ = *P*_c_ and *P*_L_ is negligible in a closed vacuum system, Eq. (1) can be expressed in detail as:

2 $$I_{{\text{W}}} \times T \times \alpha - I_{{\text{W}}}^{2} \times R - P_{{\text{h}}} = - \Delta T/R_{\text{th}}$$

where *I*_w_, *T*,* α* and *R* are the working current, absolute temperature, Seebeck coefficient and internal resistance of the μ-TCer, respectively. Hence, as a primary indicator of the μ-TCers for a power electronic component (with a heating current $I_{\text{h}}$), Δ*T* can be controlled by regulating the magnitude and direction of *I*_w_ (Fig. 2a). And its power consumption *P* can be expressed as [38]:

3 $$P = I_{{\text{w}}}^{ 2} \times R - I_{{\text{w}}} \times \Delta T \times \alpha$$

Furthermore, considering that the *P*_c_ value is always equal to the *P*_h_ value, as a comprehensive evaluation index, the $R_{\text{th}}^{\text{ e}}$ = Δ*T*/*P*_h_ defined in this study can be described as:

4 $$R_{{{\text{th}}}}^{{\text{ e}}} = \Delta T/\left({P_{{\text{P}}} {-} P_{{\text{J}}}/2 + \Delta T/R_{\text{th}}}\right)$$

According to Eq. (4), $R_{\text{th}}^{\text{ e}}$ can be regulated by the Peltier and Joule effects, which can reflect both the temperature control range and efficiency of the μ-TCers for low-power components. In addition, we systematically analysed the effect of thermal resistances (intrinsic *R*_th_ and tunable $R_{\text{th}}^{\text{ e}}$) on temperature control using a heat-compensation method (Sect. S1.2), including the temperature control capability (*η* = Δ*T*/*P*) and coefficient of performance (*COP* = *P*_c_/*P*).

## Results and Discussion

### Cooling for Ultra-Low Power Electronics

For an ultra-low power component (*I*_h_ = 5 μA and negligible *P*_h_ < 50 nW), the heating temperature difference continuously increases as a function of *I*_w_ from 0 to − 1 mA (Figs. 2a and S3d), due to the combined influence of Peltier and Joule effects [39]. Conversely, when *I*_w_ is reversed, the cooling temperature difference (Δ*T*_c_ = *T*_a_ – *T*_s_) decreases with an increase of *I*_w_ and reaches a minimum value before increasing (0–5 mA). Accordingly, Δ*T*_c_ is decisive for the temperature control range, which is also the unique feature that distinguishes TECs from microheaters with only a heating function.

For a comparative analysis of geometric parameter effect on the cooling performance (Fig. 2b), a series of devices with different section-to-length ratios (*S*/*L*) were fabricated (labelled NO.1–NO.9 in Tables S1 and S2). As the *S*/*L* value increases, the maximum cooling temperature difference $\Delta {T}_{\text{ c}}^{ \, {\text{max}}}$ increases, due to the relatively reduced effect of conductive heat loss through the Pt sensor (Fig. 1e), which was revealed by our simulations (Sect. S2 and Fig. S4). In contrast, the *R*_th_ value decreases as the *S*/*L* value increases because of their reciprocal relationship. As shown in Eq. (1), a large intrinsic *R*_th_ can suppress *P*_con_ for efficient cooling, therefore, the Δ*T*_c_ and *R*_th_ are two key parameters of the on-chip μ-TCer. Due to the small *S*/*L* of TE legs in our TCers, the ultra-high intrinsic *R*_th_ is two orders of magnitude higher than those of conventional TECs [40–42], resulting in extremely high *η* valves and greatly improved $\Delta {T}_{\text{ c}}^{ \, {\text{max}}}$ (detailed comparison in Fig. 2c and Sect. S3).

**Fig. 3:**
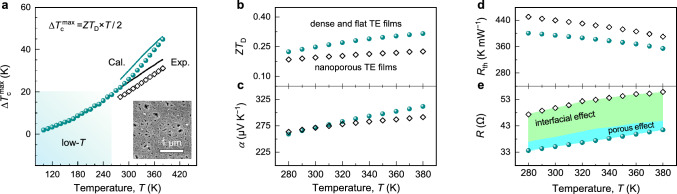
Device thermoelectric performance. Temperature-dependent **a** experimental (data point) and calculated (line) maximum cooling temperature difference $\Delta T_{{\text{c}}}^{ \max }$, **b** dimensionless device figure of merit *ZT*_D_. **c** Device Seebeck coefficient *α*, **d** thermal resistance *R*_th_ and **e** internal resistance *R* of the μ-TCers, NO.7 with dense TE films (green) and NO.9 with porous TE films (black), respectively. The inset in **a** shows the morphological details of the porous TE films

Since increasing Δ*T*_c_ and *R*_th_ to improve the temperature controllability and efficiency can not be simultaneously achieved, specific geometric parameters need to be re-designed based on practical applications. In this study, when the *S*/*L* value increases from 1.3 to 2.3 μm, the *R*_th_ and *η* valves decrease rapidly (Fig. 2b, c), but the $\Delta T_{{\text{c}}}^{ \max }$ value increases slowly. This is due to the local temperature rise of the heat sink in this single-stage TCer. Multi-stage cooling strategies (e.g., TECs combined with water/air cooling systems [43] and multi-stage TECs [44]) can overcome this bottleneck while reducing their efficiency [43]. Notably, they are not suitable as on-chip μ-TCer that require simple and compact structures. For comprehensive considerations, the appropriate *S*/*L* value is 1.5 μm (NO.7). The temperature-dependent $\Delta T_{{\text{c}}}^{ \max }$ values are shown in Fig. 3a. Specifically, it can achieve temperature differences of ~ 2 K to ~ 45 K at *T*_a_ of 100–380 K, a 100% improvement compared to flexible porous TE films with ordered microstructures [36]. For example, this on-chip μ-TCer can build and maintain a constant temperature region of 335 K for an ultra-low power component in a 380 K environment (Fig. S3e), making it quite suitable for the temperature control of modern microelectronics and even low-temperature electronics. Although *P*_L_ increases with working pressure, resulting in temperature control losses, the industrial ~ 10-mTorr vacuum packaging [45] can ensure temperature control losses of less than 0.5% (Sect. S4).

Figure 3 shows the temperature-dependent device TE performance of our μ-TCers, which were evaluated to explain the key role of device integration in their cooling ability. The cooling performance of the TCer (NO.7) fabricated by integrating dense and flat TE films is significantly higher than the TCer (NO.9) integrated by porous TE films (Fig. 3a). The experimental $\Delta {T}_{\text{ c}}^{ \, {\text{max}}}$ values for the former also match the predicted values better than the latter, based on their device figure of merit (defined as [46], *ZT*_D_ = $\alpha^{2}$×*R*_th_/*R* × *T* in Fig. 3b). In detail, the *α* values of these two μ-TCers are comparable without being significantly affected by the nanoporous structure, and increase with the increasing temperature (Fig. 3c), which is consistent with the excellent performance of our TE films (Fig. S5). However, compared with *R*_th_ (Fig. 3d), the *R* value increases more significantly by ~ 40% at 300 K, due to the interface effect (~ 30%) and porous structure (~ 10% reduction in electrical conductivity, Fig. S5), mainly leading to the decrease in cooling performance (Fig. 3a). It can be concluded that the high cooling capacity should be attributed to the high performance of freestanding TE films and their high-quality integration to reduce interfacial effects, which all benefit from the improved density and flatness of the freestanding TE films. In addition, interface engineering is expected to further optimize the interface quality, thereby improving the cooling performance [47].

### Temperature Control for Low-Power Electronics

**Fig. 4:**
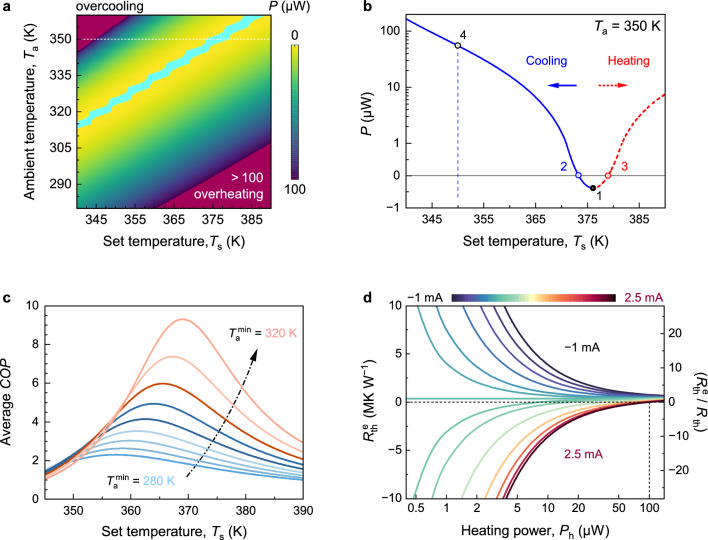
Temperature control for low-power electronics. **a** Dependence of power consumption *P* of the thermostatic control for a ~ 70-μW component on *T*_s_ and *T*_a_, detailed data in Fig. S8. **b** Power consumption *P* versus *T*_s_ (*T*_a_ = 350 K) marked by the horizontal white dotted line in **a**. **c** Average *COP* versus *T*_s_ over different temperature ranges from minimum ambient temperature ${T}_{\text{ a}}^{ \, {\text{min}}}$ to 360 K. **d** Heating power-dependent ${R}_{\text{th}}^{\text{ e}}$ and relative thermal resistance (${R}_{{{\text{th}}}}^{{\text{ e}}} /R_{\text{th}}$) under various *I*_w_ at 350 K

For efficient directionally transferring *P*_h_ of low-power electronics while maintaining a stable control temperature, *P*_c_ is as essential as $\Delta T_{{\text{c}}}$ and *R*_th_ for the μ-TCer. The *P*_c_ and *COP* of our μ-TCer exhibit a linear relationship with Δ*T* (Figs. S6 and S7), which is mainly due to the *P*_con_ shown in Eq. (1). To further evaluate the ability of our μ-TCer to stabilize the temperature for a ~ 70-μW electronic component in a variable temperature environment, we comprehensively analysed its power consumption *P* calculated by Eq. (3). Figure 4a shows the *P* of the μ-TCer as a function of *T*_s_ and *T*_a_, where the cyan area represents that *P* is less than zero, owing to its TE generation function based on the Seebeck effects [39]. The upper and lower areas close to the cyan area represent the *P* of TE cooling and heating, respectively. Figure 4b shows the *P* as a function of *T*_s_, reaching its lowest *P* at a *T*_s_ of ~ 376 K (point 1, the intersection of cooling and heating *P* curves). The Δ*T* of ~ 26 K (between this *T*_s_ of ~ 376 K and *T*_a_ of 350 K) is caused by the ~ 70-μW heating effect of the component. And driven by this Δ*T*, this μ-TCer achieved a maximum TE power generation of 0.5 μW (comparable to the on-chip TE generators [48]), leading to a minimum *P* of − 0.5 μW. When this power is used for cooling or heating, *P* is zero, marked by the points 2 and 3 in Fig. 4b, respectively. To completely transfer the 70 μW *P*_h_, a 55 μW *P* is required to eliminate Δ*T* (*T*_s_ = *T*_a_ = 350 K at point 4), resulting in a *COP* of 1.36. When *T*_s_ is below ~ 345 K, *P* increases rapidly with Δ*T* (*T*_a_ increases and/or *T*_s_ decreases) and even exceeds 100 µW, because of the overcooling in temperature (upper red area in Fig. 4a). Similarly, as the heating Δ*T* increases, *P* also increases rapidly because of the overheating (lower red area in Fig. 4a), which is consistent with the *COP* data in Figs. S7 and S8.

For the same *P*, the cooling temperature range is notably smaller than the heating temperature range due to the counteraction and combination of the Peltier and Joule effects (Fig. 4a). Therefore, an appropriate *T*_s_ is the key to realizing efficient temperature control for low-power electronics. To obtain the optimal *T*_s_, the variation of the average *COP* (${\overline{COP}}$) with *T*_s_ was summarized in Fig. 4c. In a variable temperature environment (from the minimum ambient temperature ${T}_{\text{ a}}^{ \, {\text{min}}}$ to 360 K), all ${\overline{COP}}\text{s}$ increase as *T*_s_ increases and reach their respective maximum values before starting to decrease. For instance, when the temperature range is from 280 to 360 K (${T}_{\text{ a}}^{ \, {\text{min}}}$ = 280 K), the lowest average *P* is ~ 30 μW (Fig. S8f), leading to an ideal ${\overline{COP}}$ of 2.3 (Fig. 4c), which is comparable to the *COP*s of most air conditioners. Moreover, as the ${T}_{\text{ a}}^{ \, {\text{min}}}$ increases from 280 to 320 K, the maximum ${\overline{COP}}$ value increases and reaches as high as 9 in spite of the increase of optimal *T*_s_ by 10 K (Fig. 4c). Importantly, the ${\overline{COP}}$ of 6 could be achieved in wearable and implantable electronics because their *T*_a_ is always over 310 K, pointing us in a promising practical direction for our μ-TCers.

This μ-TCer aims at building a large and controllable ${R}_{\text{th}}^{\text{ e}}$ to minimise passive *P*_con_ and achieve efficient temperature control for low-power microelectronics. Figure 4d shows the results of the ${R}_{\text{th}}^{\text{ e}}$ value calculated by Eq. (4) as a function *P*_h_ under various *I*_w_ at a *T*_a_ of 350 K, the ${R}_{\text{th}}^{\text{ e}}$ value decreases significantly as *I*_w_ increases and can be zero in cooling mode, indicating that the *P*_h_ of the microelectronic was transferred through the Peltier and Thomson effects without forming any Δ*T*. In particular, when *I*_w_ is zero, the ${R}_{\text{th}}^{\text{ e}}$ value is ~ 350 K mW^−1^, which is also the intrinsic *R*_th_. As *I*_w_ continues to increase, the ${R}_{\text{th}}^{\text{ e}}$ value becomes negative, this is the unique feature of the TE effect – transferring heat from the low-temperature side to the high-temperature side. For a ~ 5-μW component, the ${R}_{\text{th}}^{\text{ e}}$ value can be adjusted arbitrarily within the range of ± 7.5 MK W^−1^ (more than ± 20 times compared to intrinsic *R*_th_). However, it can be seen that the lower the *P*_h_ of the microelectronic, the better the temperature controllability, which makes the μ-TCer more suitable for low-power electronics.

The widely controllable ${R}_{\text{th}}^{\text{ e}}$ is critical for this μ-TCer to achieve highly energy-efficient temperature control, including the multi-levels of energy savings: (1) micro-zone temperature control; (2) ultra-high thermal resistance; (3) a combination of TE cooling, heating and generation. The high intrinsic $R_{\text{th}}$ is mainly due to the larger geometry controllability of the *S*/*L* (Fig. 2b) compared to other integrated TE devices [48] and the low thermal conductivity of in-plane TE films, as well as avoiding heat loss from substrates [42] and gas environment (Supplementary Sect. 4). The wide tunability of ${R}_{\text{th}}^{\text{ e}}$ relies on the superior *ZT*_D_ value (Fig. 3b) and the high-quality integration of dense TE films in the compact unicouple μ-TCers to reduce interfacial effects.

### Controllability and Stability

**Fig. 5:**
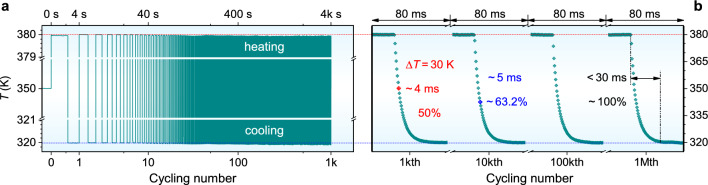
Controllability and stability of the micro TE TCer. Cycling number-dependent control temperature results of the micro TE TCer using an alternating working current (− 0.5/1.85 mA) for heating and cooling, respectively. **a** Continuous test results (2 s heating and 2 s cooling). **b** Intermittent test results during a single cycle (~ 80 ms cooling)

To evaluate the reliability of the μ-TCer, we investigated the temperature control stability and cycling durability using an alternating *I*_w_ to drive the cooling and heating functions. Figure 5a shows that during the continuous test (including cooling and heating), the control temperature fluctuates by less than 0.1 K per cycle, and less than 0.2 K after 1000 cycles, demonstrating excellent operational stability. Note that the overall temperature variation throughout 1 h mainly comes from the fluctuating *T*_a_ instead of the changes in the control temperature, confirmed by the differences and average values of cooling and heating temperatures (Fig. S9). After excluding the effect of *T*_a_ fluctuations, the relative temperature changes of continuous operation is about 0.4% – an order of magnitude improvement over our previously reported highest value [32].

The effect of cycling number on control temperature was also characterized to further assess cycling durability and response time (Fig. 5b). The test period of ~ 200 ms is much longer than the time constant of the Peltier and Joule effects [49] to ensure test accuracy. The temperature change is still less than 0.2 K even after 1 million (M) cycles, showing good temperature control stability and reliability. The μ-TCer takes only ~ 4 ms to cool the heating zone from 380 to 350 K (*T*_a_) and features a short response time of 5 ms to further reduce the temperature to 342 K (63.2%), and a total of 30 ms is enough to stabilize the temperature at 320 K during the whole 1 M cycles. This response time is comparable with that of the out-of-plane micro TECs [50] and several orders of magnitude shorter than that of traditional bulk TECs for commercial and academic research [51]. The average cooling rate of 2000 K s^−1^ is three orders of magnitude higher than the bulk TE coolers [33, 50], owing to its small thermal capacity and high cooling power density.

There are four factors mainly contributing to the excellent reliability: (1) the freestanding flexible films have good flexibility [52] and thus can absorb thermal stress or strain [32], thereby improving structural stability; (2) the simple unicouple structure makes its series circuit a lower risk of failure than a TEC containing hundreds of TE legs; (3) in contrast to the ampere-level *I*_w_ reported in the superlattice film-based [41, 53] or bulk [54, 55] TECs, the low *I*_w_ (~ 3 mA) has the advantage of generating less heat, as well as avoiding potential breakdown effects and electromagnetic interference on nearby electronics; (4) the vacuum packaging environment minimizes the chemical degradation of device material, thereby improving TE performance stability. Therefore, this μ-TCer can be used for precise temperature control of low-power temperature-sensitive electronics. It can also make an important contribution to the modulation of frequency for on-chip lasers [11, 12] and MEMS clocks [13, 14].

## Conclusions

We report an on-chip micro temperature controller consisting of a unicouple of dense and flat freestanding TE films deposited on a newly developed ultra-thin GO nano membrane and further demonstrate its implementation in micro-zone thermal management for low-power temperature-sensitive microelectronic components. It combines the features of large intrinsic *R*_th_ and widely tunable ${R}_{\text{th}}^{\text{ e}}$ from positive to negative, leading to energy-efficient temperature control (${\overline{COP}}$ > 2.3 and *η* > 100 K mW^−1^). The combination of Joule and Peltier effects gives it an ultra-fast cooling rate (2000 K s^−1^) and outstanding cooling temperature difference (~ 45 K). An extremely compact device structure combined with superior reliability (> 1 M) could be important guarantees for its practical applications. In conclusion, we established an unprecedented design concept, conducted temperature control analyses of the prototype devices, proposed ${R}_{\text{th}}^{\text{ e}}$ as a comprehensive performance evaluation indicator. We hope that such temperature controllers can overcome emerging challenges in the ever-developing energy-efficient microelectronics.

## Supplementary Information

Below is the link to the electronic supplementary material.

Supplementary file1 (PDF 5773 KB)
