# Supplementary material for: On-Chip Micro Temperature Controllers Based on Freestanding Thermoelectric Nano Films for Low-Power Electronics
Source: Nanomicro Lett. 2024 Feb 20;16:126. doi: 10.1007/s40820-024-01342-3 (PMC10879069; doi:10.1007/s40820-024-01342-3)
Supplement: Supplementary file 1 — Supplementary file1 (PDF 5773 KB) [file 40820_2024_1342_MOESM1_ESM.pdf]

Supporting Information for

On-Chip Micro Temperature Controllers Based on Freestanding Thermoelectric Nano Films for Low-Power Electronics

Qun Jin[1, *], Tianxiao Guo[2], Nicolás Pérez[1], Nianjun Yang[2], Xin Jiang[2], Kornelius Nielsch[1, 3, 4, *] and Heiko Reith[1, *]

[1]Institute for Metallic Materials, Leibniz Institute for Solid State and Materials Research Dresden, Dresden 01069, Germany

[2]Institute of Materials Engineering, University of Siegen, Siegen 57076, Germany

[3]Institute of Applied Physics, Technical University of Dresden, Dresden 01069, Germany

[4]Institute of Materials Science, Technical University of Dresden, Dresden 01069, Germany

*Corresponding authors. E-mail: q.jin@ifw-dresden.de (Qun Jin); k.nielsch@ifw-dresden.de (Kornelius Nielsch); h.reith@ifw-dresden.de (Heiko Reith)

Section S1 Preparation and Characterization Methods

S1.1 Fabrication of ultra-thin freestanding thermoelectric (TE) films

First, the single-walled carbon nanotube (SWCNT) CNT films prepared by vacuum filtration of CNT solution were transferred onto a SiN/Si scaffold, the optical images shown in Fig. S2a, b. A commercial aqueous SWCNT suspension with an average diameter of 1.5 nm, lengths of ~15 μm and >95% purity were used (Jiangsu XFNANO Materials Tech Co., Ltd). A well-dispersed ~300 ml SWCNT suspension was obtained after 1 h ultra-sonication and 30 min centrifugation, and then was vacuum filtrated with a cellulose membrane with a pore size of 450 nm to form a CNT film, as shown in Fig. S2c, the top-view SEM image of prepared CNT film exhibits very good uniformity. Then ultra-thin hybrid membranes were prepared by pulling the CNT films from the monolayer graphene oxide (GO) solution after ultrasonic dispersion (~1 mg GO nanosheets in 10 mL deionized water, XFNANO Materials Tech Co., Ltd), which were shown in Fig. S2d (pulling process) and Fig. S2e (SEM image, showing the underlying CNTs under electron beam through ultrathin translucent GO). Figure S2f–j shows the SEM images of ~750 nm p- and n-type freestanding TE films on hybrid membranes deposited by magnetron sputtering, showing good flatness and high density. The microstructures and phase purity of the high-quality samples were characterized by XRD and SEM, and EDS were used to characterize the compositions of the samples (Figs. S2, S10 and S11). The thicknesses of the TE films were measured by cross-sectional SEM images (details in Fig. S2g, i).

S1.2 Integration process and measurement methods of TCers

The fabrication process of the on-chip TCers is shown in Fig. S1, standard MEMS process technologies were used to fabricate the suspended Si_3N_4 layer and multilayers of test electrodes in the device (**Section 2.3** in main text). Finally, the n- and p-type freestanding TE films were integrated onto the above-prepared chips by the focused dual-beam (e/Ga) technique, including patterned cutting, transferring and bonding. C-layer deposition by e-beam before cutting and bonding by Ga-ion-beam can effectively prevent Ga ion implantation or diffusion into TE films from affecting the TE performance. In addition, the oblique cutting section is to increase the contact area to reduce contact resistance and thermal resistance. The integrated micro TE TCer is shown in the SEM image (Fig. 1e). The PPMS system can provide a high vacuum environment (~0.01 mTorr) and accurate setting ambient temperature (T_a, from 280 to 380 K, similar to the operating temperature range of microelectronics) for performance test of our μ-TCers.

1) ***Temperature and temperature difference*** – A Pt temperature sensor was used to assess the temperature controllability of our μ-TCers by monitoring its resistance value in real-time (as shown in Fig. S3a). Based on the linear relationship between the Pt resistance (R_{Pt}) and temperature (Fig. S3b), we can obtain accurate set temperature T_s (Fig. S3c) and temperature difference $\Delta T = T_s - T_a$ (Figs. S3d and 2a).

2) ***Cooling power*** – Heat-compensation method was proposed to directly test the cooling power (P_c) and coefficient of performance (*COP*, cooling power divided by the electrical power consumption) of our μ-TCer rather than the estimation based on material properties [S1-S3], in which, the key heating current I_h can simulate the Joule heating effect of the micro component (heating power, $P_h = I_h^2 \times R_{Pt}$) and simultaneously monitor the real-time temperature (see Fig. 1 for the schematic diagram, detailed data in Fig. S6a. For example, the relationship between the temperature control value and the two currents are shown in Fig. S6b (*xz*-projection of Fig. 2a) and Fig. S6c (*yz*-projection of Fig. 2a). Thus, by adjusting the heating power, the cooling power test under the condition of constant ΔT can be realized ($P_c = P_h$), including thermoelectric cooling power when ΔT is 0 K (Fig. S7a, c) based on the critical conditions shown in Fig. S6d and the cooling power under different ΔTs (Fig. S7b).

3) ***Thermoelectric performance of micro TE TCers*** – The internal resistance *R* can be tested in PPMS at different ambient temperatures T_a. In addition, the voltage *V* of the Pt sensor and Seebeck voltage U_s of the μ-TCer as a function of I_h could be tested (Fig. S6e), as well as corresponding heating power ($P_h = V \times I_h$) and $\Delta T = T_s - T_a$ (Fig. S6f, g). The approximately linear relationship of P_h and U_s with ΔT can be used for calculating the thermal resistance ($R_{th} = \Delta T / P_h$), and device Seebeck coefficient ($\alpha = U_s / \Delta T$) , which includes the Seebeck coefficient of a pair of the P- and N-type thermoelectric legs, ($\alpha = \alpha_P - \alpha_N$) of the μ-TCer, respectively, detailed data are shown in Fig. 3.

4) ***Efficiency measurement*** – The *COP* can be obtained by dividing the P_c by the power consumption (*P*), as shown in Fig. S7d, e. Note that power consumption can be obtained by calculating, $P = (I_w^2 \times R - I_w \times \alpha \times \Delta T)$, and temperature control capability η can be obtained by calculating, $\eta = \Delta T / P$ (Fig. 2c). Thus, we can also test the temperature control performance of simulated power electronics (Fig. 4) based on the detailed data in Fig. S8. When T_s is set as 360 K, based on the curve of *P* with T_a (Fig. S8e), the calculated average power consumption $\overline{P}$ is only ~32 μW after avoiding overcooling and overheating (Fig. S8f).

Section S2 Conductive Heat Loss through the Pt Sensor

Basically, the *S*/*L* value directly affects the internal resistance *R* and thermal resistance R_{th} of the TE legs, and therefore indirectly affects the total internal resistance $R_{tot} = R + R_{in}$ (*R* and interface resistance R_{in} are in series) and total thermal resistance $R_{th}^{tot} = R_{th} + R_{th}^{in}$ (R_{th} and interface thermal resistance R_{th}^{in} are in series) of the TE devices (Tables S1 and S2).

In the out-of-plane film TE device, since the *S*/*L* value is relatively large compared with in-plane films (Table S3), the proportion $\lambda_1 = R/R_{tot}$ and $\lambda_2 = R_{th}/R_{th}^{tot}$ are not high enough. In order to reduce the adverse effects of R_{in} and R_{th}^{in} by increasing λ_1 and λ_2 valves, R_{th} and *R* can be increased by extending the *L* value (or reducing *S*/*L*), which can increase the cooling temperature difference ΔT_c (ref. [S4]). When *L* increases and the proportion λ is close to 100%, the theoretical maximum ΔT_c (ΔT_{c0}^{max}) could be achieved, which is then mainly determined by TE properties if there is no other heat loss.

On the contrary, the *S*/*L* values of our in-plane films are already very small (two orders of magnitude lower than the out-of-plane films, see Table S3), and λ should be close to 100%. For only TE legs, the relationship between cooling power P_c with ΔT_{c0}^{max} and G_{th} (thermal conductance G_{th} =

$1/R_{th}$) can be approximately expressed as: $P_c = G_{th} \times \Delta T_{c0}^{max}$. Then when considering the conductive heat loss through the Pt sensor (its G_{th*} is in parallel with G_{th}). Therefore, $P_c = G_{th}^{tot} \times \Delta T_c^{max}$ and $\Delta T_c^{max} = (\Delta T_{c0}^{max} \times G_{th})/(G_{th} + G_{th*})$, details in Table S2. As a result, the factors that actually affect the ΔT_c^{max} are the heat loss (G_{th*}) and the *S*/*L* value since G_{th} is linear with the *S*/*L* value. The conductive heat loss through the Pt sensor is the main reason why ΔT_c increases with the increasing *S*/*L* value (Fig. 2b).

To further verify the above results, we performed Finite Element Simulation using the COMSOL Multiphysics software. The device design parameters and materials properties can be found in Tables S1 and S2 and Fig. S5. First, the thermal conductivities (κ) of the thermoelectric thin films are calculated using the Wiedemann-Franz law ($\kappa = L\sigma T + \kappa_l$), the Lorenz number $L = 2.4\times10^{-8}$ V^2 K^{-2} (ref. [S5]), the electric conductivity (σ) as a function of absolute temperature (T) can be found in Fig. S5. To match the thermal resistance value R_{th}^{tot} of TCer in Fig. 3d, the lattice thermal conductivity (κ_l) is set as 0.45 (0.4) W m^{-1} K^{-1} for p (n)-type film. And considering the influence of the interface effect, the tuning factor F (defined as the ratio of the performance used in device simulation to the material performance), F1 = 0.8, F2 = 0.9 and F3 = 0.9 are used for the Seebeck coefficient, electric and thermal conductivities, respectively. The simulation results are shown in Fig. S4, the maximum cooling temperature difference increases but the rate of increase gradually decreases with the increase of the *S*/*L* valve. These results directly indicate that conductive heat loss is an important factor affecting the cooling temperature difference. The calculated heat loss ratios data of each device are shown in Table S2.

Section S3 Cooling Performance Comparison

From the viewpoint of application for microsystem temperature control, one of the advantages of micro thin-film (out-of-plane and in-plane) TECs (http://www.lairdtech.com) is that they feature higher cooling power density compared to bulk TECs (https://rmtltd.ru) and are therefore considered to be more suitable for thermal management of microsystems. The ultra-high R_{th} of our μ-TCer is ~345 K mW^{-1}, which is two orders of magnitude higher than that of the out-of-plane TECs due to the smaller *S*/*L* of our TE legs, and also higher than that of non-freestanding in-plane TECs by avoiding heat loss from the substrates (Fig. 2c and Tables S1 and S3), making its temperature control capability η extremely high (Fig. S8d). The η values are several orders of magnitude higher than those of conventional TECs and microheaters [6], exceeding 100 K mW^{-1} even under the maximum cooling temperature ΔT_c^{max}. The η values of our μ-TCers increase significantly with increasing R_{th} (with decreasing *S*/*L* value from NO.7 to NO.1 and/or ambient temperature from 380 K to 300 K), which are shown in Fig. 2c. Therefore, we can conclude that large R_{th} is extremely important for energy-efficient temperature control. In addition, the achievable cooling temperature differences of our in-plane μ-TCers are much higher than those of previously reported in-plane TECs, and comparable to the best out-of-plane superlattice-based TECs (details in Tables S1 and S3). We compared the ΔT_c^{max} and the maximum cooling power P_c^{max} values of our μ-TCer with those of traditional thin-film TECs in Fig. S13. While the cooling power of in-plane TECs is only sub-mW much lower than that of out-of-plane devices due to the extremely small cross-sectional area, which makes the former more suitable for low-power micro components, and the latter for high-power devices in thermal management.

Section S4 Convective and Radiative Heat Loss

Figure S12 shows that increasing working pressure results in a non-negligible heat loss power P_L through air convection, ultimately leading to a 95% reduction in ΔT_c at atmospheric pressure. Even when the pressure decreases to ~7 Torr, the ΔT_c and heat loss values also change rapidly, which indicates that heat transfer at the solid-gas interface is comparable to the heat conduction through TE legs. The performance of the microheater [S7] and microfluidic calorimeter [S8] are also sensitive to

this Torr-scale pressure. Fortunately, vacuum packaging can meet the working pressure requirement, the performance reduction is less than 0.5% in an industrial 10-mTorr vacuum [S9].

Table S1 | Table of all design parameters and performance of TE TCers

			Device number								
**Parameter**		**Unit**	NO.1	NO.2	NO.3	NO.4	NO.5	NO.6	**NO.7#**	NO.8	NO.9##
**Length**	**L**	(μm)	50	40	40	40	30	30	**25**	30	25
**Width**	**W**	(μm)	50	42	50	56	50	54	**50**	45	50
**Height**	**H**	(μm)	0.75	0.75	0.75	0.75	0.75	0.75	**0.75**	1.5*	0.75
**Width/Length**	**W/L**	(-)	1	1.05	1.25	1.4	1.67	1.8	**2**	1.5	2
**S/L**	**W*H/L**	(μm)	0.75	0.79	0.94	1.05	1.25	1.35	**1.5**	2.25	1.5
**Maximum cooling power**	P_c^{max}	(μW)	53.4	58.5	71.3	82.5	103.4	115.5	**130**	188.4	94
**Maximum ΔT**	ΔT_c^{max}	(K)	30	31	34.5	37.9	41.8	44	**44.7**	45.4	31
**Power density**	P_c^{max}/S	(W cm^{-2})	71.2	92.9	95.1	98.2	137.9	142.6	**173.3**	139.6	125.3
**ΔT-gradient**	$\Delta T_c^{max}/L$	(K mm^{-1})	600	775	863	948	1393	1467	**1788**	1513	1240
**Thermal resistance****	R_{th}	(K mW^{-1})	561.8	529.9	483.9	459.4	404.3	381.0	**344**	241.0	390
**Resistance**	**R**	(Ω)	71.1	70.1	62.3	55.0	50.1	45.2	**39.5**	30.0	54.1
**Material Resistance**	R_m	(Ω)	60.1	57.1	49	42.8	36.2	33.3	**30.1**	20.2	33.1
**Optimal current**	**I**	(mA)	1.60	1.68	1.96	2.25	2.55	2.84	**3.12**	5.00	2.4
**Power consumption**	**P**	(μW)	196.4	213.5	259.6	304.0	357.8	402.1	**430**	818.1	330
**Temperature control capability** η	$\Delta T_c^{max}/P$	(K mW^{-1})	152.7	145.2	132.9	124.7	116.8	109.4	**103.9**	55.5	94

# Unless otherwise specified, TCer refers to NO.7 in this work. ## NO.9 TE TCer with nanoporous TE films.

* Double layer thermoelectric film. ** Thermal resistance R_{th} is defined as $\Delta T_c^{max}/P_c^{max}$.

*** All cooling performance at 380 K (in PPMS system).

Table S2 | Table of materials properties and geometry parameters (Pt/SiO_2/Si_3N_4/Au layers)

**Parameter**			**Unit**	**Device number**								
				**NO.1**	**NO.2**	**NO.3**	**NO.4**	**NO.5**	**NO.6**	**NO.7**	**NO.8**	**NO.9**
**Length**	**Pt/SiO_2/Si_3N_4**	**L1**	(μm)	260	260	280	280	300	300	300	300	300
	**Au**	**L2**	(μm)	120	120	120	120	120	120	120	120	120
**Width**	**Pt**	**W1**	(μm)	16	16	16	16	16	16	16	16	16
	**SiO_2/Si_3N_4**	**W2**	(μm)	30	30	30	30	30	30	30	30	30
	**Au**	**W3**	(μm)	100	100	100	100	100	100	100	100	100
**Thickness**	**Pt**	**D1**	(nm)	32	32	38	40	40	38	38	38	38
	**SiO_2**	**D2**	(nm)	80	80	50	60	60	50	50	50	50
	**Si_3N_4**	**D3**	(nm)	100	100	100	100	100	100	100	100	100
	**Au**	**D4**	(nm)	180	180	180	180	180	180	180	180	180
**Thermal conductivity**	**Pt**	***40***	(W m^{-1} K^{-1})									
	**SiO_2**	***3***	(W m^{-1} K^{-1})									
	**Si_3N_4**	***4***	(W m^{-1} K^{-1})									
	**Au**	***200***	(W m^{-1} K^{-1})									
**Electric conductivity**	**Au**	**2.28e7**	(S m^{-1})									
**Thermal resistance**	**TE TCer**	R_{th}^{tot}	(K mW^{-1})	561.8	529.9	483.9	459.4	404.3	381.0	343.8	241.0	390
**Thermal conductance**	**TE TCer**	G_{th}^{tot}	(μW K^{-1})	1.78	1.887	2.067	2.177	2.474	2.625	2.908	4.159	2.564
**Thermal conductance**	**Pt sensor**	G_{th*}	(μW K^{-1})	0.610	0.610	0.583	0.614	0.573	0.544	0.544	0.544	0.544
**Thermal conductance**	**TE legs**	G_{th}	(μW K^{-1})	1.170	1.277	1.484	1.563	1.901	2.081	2.364	3.615	2.02
**Maximum ΔT**	**TE TCer**	ΔT_c^{max}	(K)	30	31	34.5	37.9	41.8	44	44.7	45.4	31
**Heat loss ratio of Pt sensor**	-	G_{th*}/G_{th}^{tot}	(%)	34.30	32.35	28.21	28.22	23.17	20.73	18.72	13.09	21.21

[#] G_{th*} of four legs of Pt sensor can be evaluated based on geometry parameters and material thermal conductivities at 380 K.

Table S3 | Selected references for the comparison of TECs used in Fig. 2c

			References								
Parameter		Unit	[S1]	[S2]	[S10]	[S11]	[S12]	[S13]	[S14]	[S15]	Our work
Length	L	(μm)	50	10	2.3k *	8.1	20k *	20	1.6k	20k *	25
Width	W	(μm)	50	30	1.5k	-	4k	-	1.6k	4k	50
Diameter	D	(μm)	-	-	-	130/180 /200/230 ***	-	60	-	-	-
Height (N+P)	H	(μm)	1.37	145	2.15	-	110	-	3.2k	50	1.5
Area (N+P)/2	S	($μm^2$)	34.25	2.2k	3.2k	13k/25k /31k/41k	220k	2.8k	2.6M	200k	37.5
S/L	S/L	(μm)	0.7	218	1.4	1.6k/3.1k /3.8k/5K	11	141	1.6k	10	1.5
Temperature	T	(K)	320/360 /400 ***	290	300	298	300	355	304	300	300/320/340 /360/380 ***
Maximum cooling power	P_c^{max}	(μW)	49/57 /63	2.5k	0.14	21k/47k /67k/107k	616	30	3.4k	850	68/80/95 /111/130
Maximum ΔT	ΔT_c^{max}	(K)	16.4/19.8 /22.5	6.3	0.2	45.6/40.5 /36/43.5	~0.8	2	8.8	0.7	25.9/30/34.7 /39.7/44.7
Power density	P_c^{max}/S	($W\ cm^{-2}$)	72/84 /92	57	4.2	158/184 /214/258	0.14	7	0.13	0.45	91/107/127 /148/173
ΔT-gradient	ΔT_c^{max} / L	($K\ mm^{-1}$)	328/396 /450	630	0.1	5.6k/5k /4.4k/5.4k	0.04	0.1	5.5	0.03 5	1k/1.2k/1.4k 1.6k/1.8k
Thermal resistance **	R_{th}	($K\ mW^{-1}$)	334/347 /357	2.5	1.4k	2.2m/0.9 m 0.5m/0.4 m	1.3	67	2.6	0.82 4	380/376/365 /358/344
Resistance	R	(Ω)	268/285 /301	1.05	63	0.00687	5	0.26	0.07 5	1.67	39.5 (380 K)
Optimal current	I	(mA)	0.85/0.93 /1	70	0.1	10k/12k/ 14k/14.8k	20	100	2.5k	60	3.12 (380 K)
Power consumption	P	(μW)	194/245 /300	5.1k	0.63	///1.5M	2k	2.6k	78k	6k	430 (380 K)
Temperature control capability	ΔT_c^{max} / P	($K\ mW^{-1}$)	84.5/80 /75	1.24	159	0.03	0.4	0.77	~0.1	0.12	103.9 (380 K)

[#] All cooling performance for unicouple devices unless otherwise specified. * Single leg device.

** Thermal resistance R_{th} is defined as $\Delta T_c^{max}/P_c^{max}$. *** Different geometric parameters or temperatures.

Fig. S1 Integration of on-chip micro thermoelectric TCer

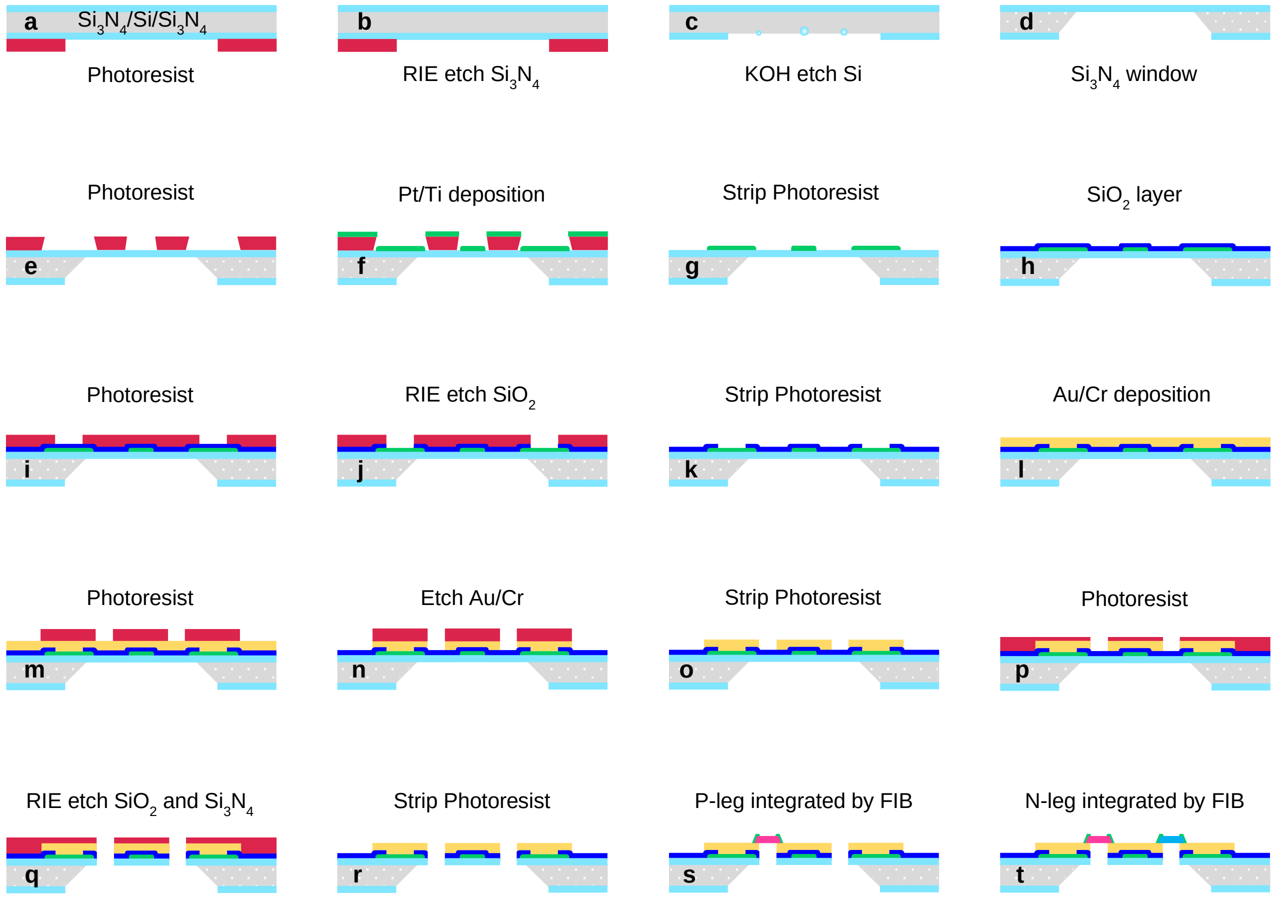

Fig. S1 Fabrication process of the on-chip micro thermoelectric temperature controller. **a** Spin-coating and patterning (SP) photoresist on ~300 µm silicon (Si) wafer with double-sided ~100 nm silicon nitride (Si_3N_4) layers. **b** Reactive Ion Etching (RIE) Si_3N_4 layer with CF_4 gas. **c** KOH Etching Si after Photoresist removal. **d** Obtained freestanding Si_3N_4 window. **e** SP photoresist on Si_3N_4 window. **f**, **g** Deposited ~44 nm Pt/Ti electrodes by magnetron sputtering before and after the lift-off process. **h** Deposition of ~50 nm SiO_2 layer by ALD. **i** SP photoresist on SiO_2 layer. **j** RIE SiO_2 layer with CF_4 gas. **k** Exposed Pt electrodes for performance testing after photoresist removal. **l** Deposition of ~200 nm Au/Cr layers by magnetron sputtering. **m** SP photoresist on Au/Cr layers. **n**, **o** Solution etching Au/Cr electrodes before and after the lift-off process. **p** SP photoresist. **q**, **r** RIE SiO_2 and Si_3N_4 layers with CF_4 gas before and after photoresist removal. **s**, **t** Integration of p-type and n-type thermoelectric legs onto above-fabricated MEMS-based chip by focused ion beam, details are provided in Supplementary Section 1.

Fig. S2 Fabrication of freestanding hybrid membranes and TE films

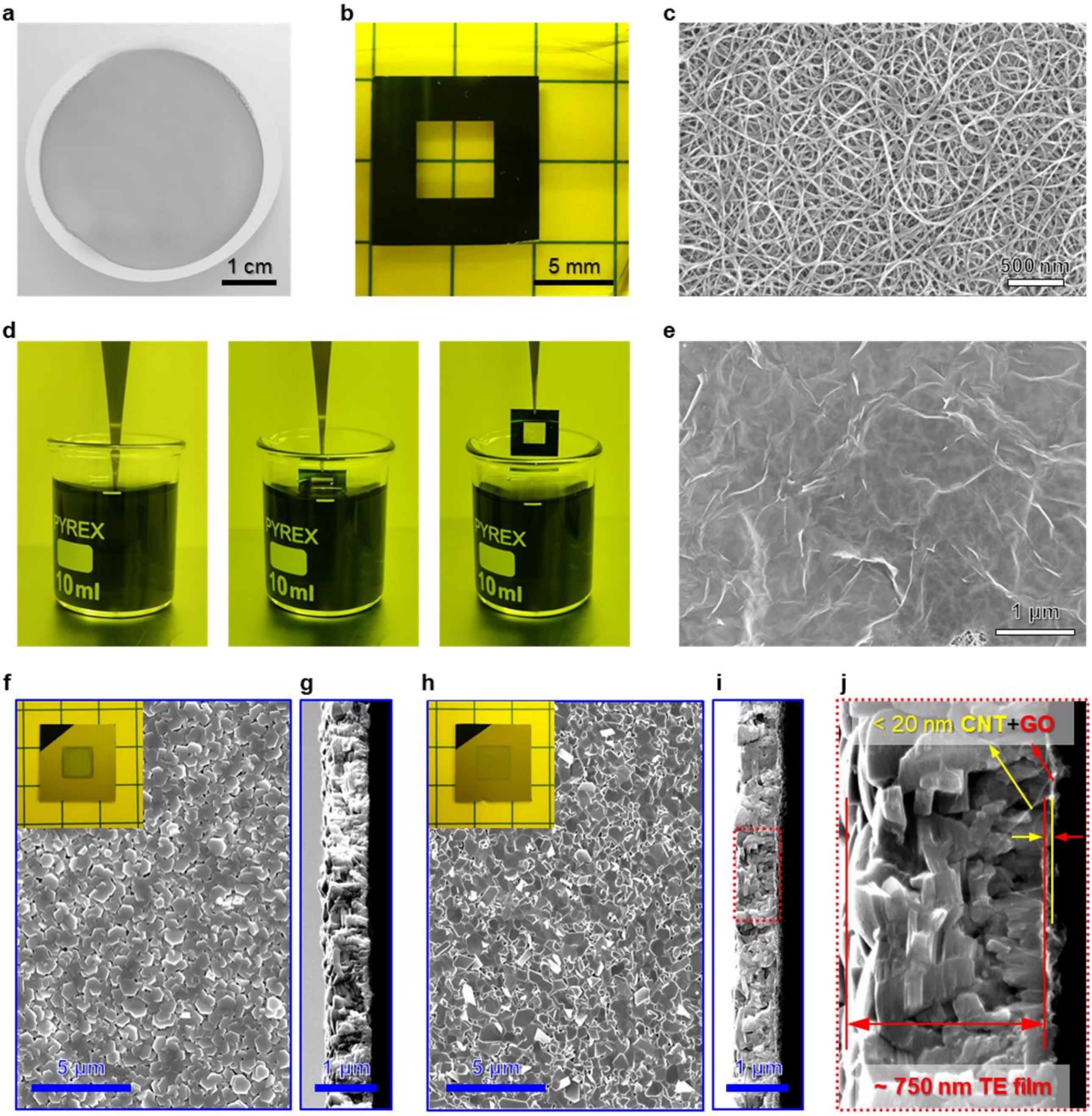

Fig. S2 Fabrication of freestanding ultra-thin hybrid membranes and thermoelectric films. **a**, **b** Optical images of single-walled carbon nanotube (SWCNT) films prepared by vacuum filtration and the transferred SWCNT films on a Si scaffold, respectively. **c** Top-view SEM image of prepared CNT film. **d**, **e** Dip-coating process and microstructure of ultra-thin hybrid membranes of monolayer graphene oxide (GO) and CNT, respectively. **f**–**i** Top-view (**f**, **h**) and cross-section (**g**, **i**) SEM images of ~750 nm p- and n-type freestanding thermoelectric films deposited by magnetron sputtering, respectively, insets are the corresponding optical pictures. **j** Close-up SEM image marked by red dotted box in **i** showing the ultrathin CNT-GO hybrid membrane (<20 nm) in detail

Fig. S3 Measuring temperature

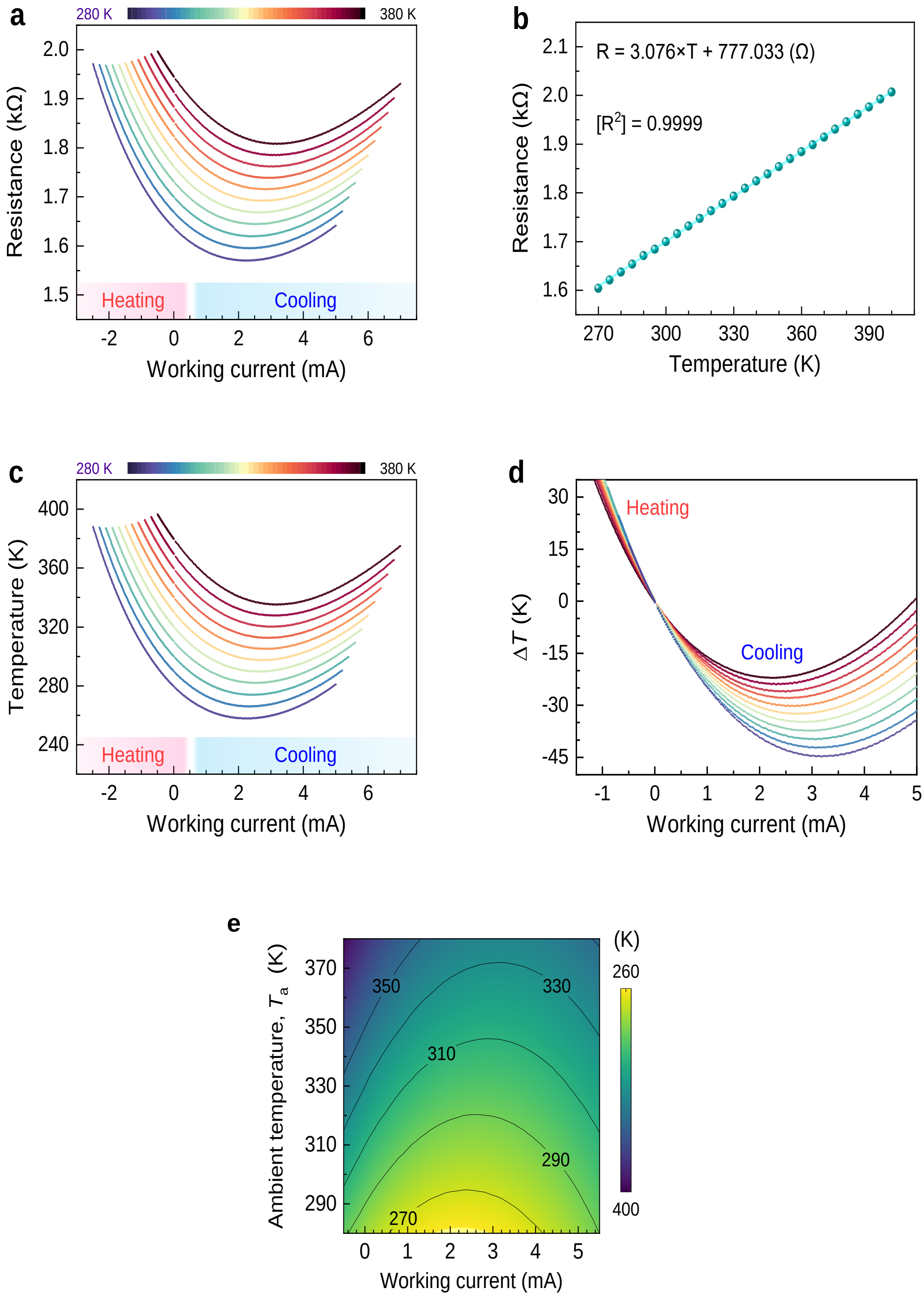

Fig. S3 Temperature controlling measurement via Pt temperature sensor. **a** Real-time resistance value of the Pt temperature sensor at different ambient temperatures (280−380 K) and working current. **b** Linear relationship between temperature and resistance of Pt temperature sensor. **c**, **d** Real-time current-dependent temperatures and temperature differences ΔT at different ambient temperatures and working current, details are provided in Supplementary Section 1.2. **e** Working current dependence of controllable constant set temperature T_s under variable ambient temperature

Fig. S4 COMSOL simulating results

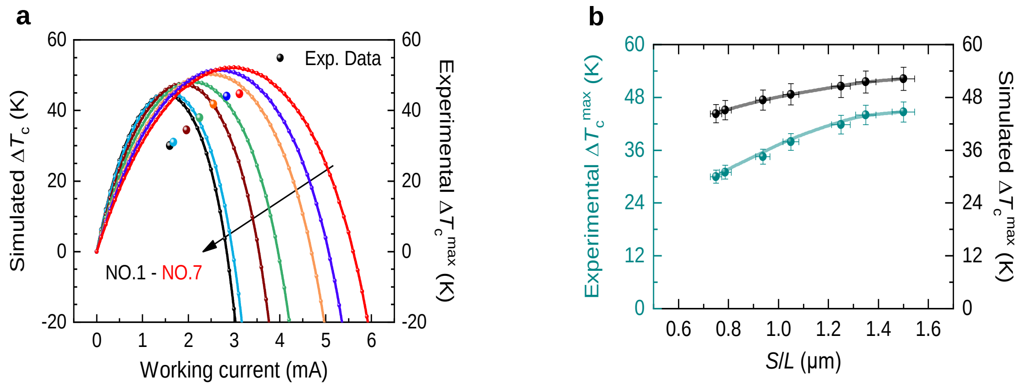

Fig. S4COMSOL simulating results. **a** Working current-dependent simulated and experimental maximum cooling temperature differences for μ-TCers (NO.1–NO.7) at T_a of 380 K and corresponding maximum cooling temperatures as a function of the *S*/*L* in a series of μ-TCers (**b**)

Fig. S5 Thermoelectric properties

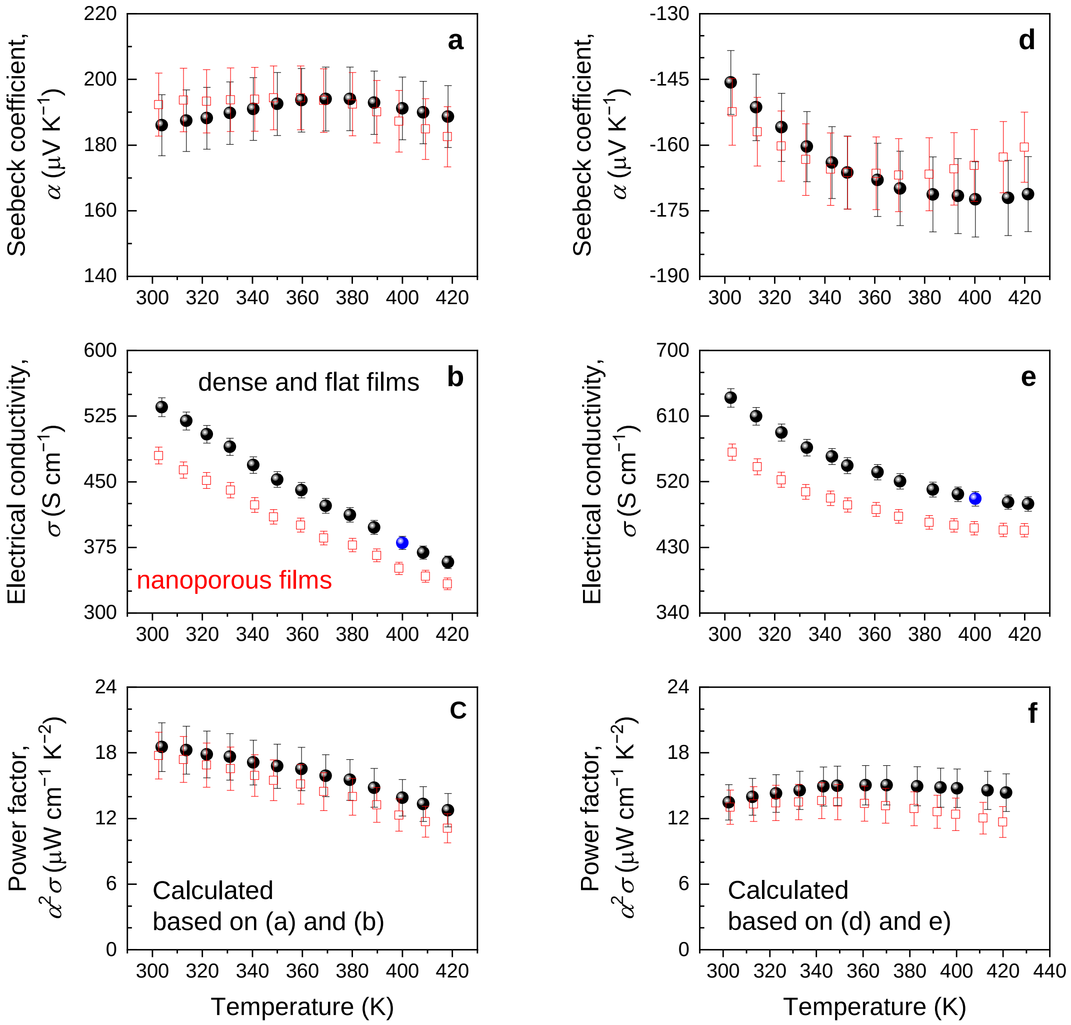

Fig. S5 Thermoelectric properties of n-type and p-type dense and nanoporous TE films, respectively. **a**, **d** Seebeck coefficient. **b**, **e** Electrical conductivity. **c**, **f** Power factor. The respective measurement uncertainties for Seebeck coefficient, Electrical conductivity and Power factor are about 5%, 2%, and 12%

Fig. S6 Heat-compensation method

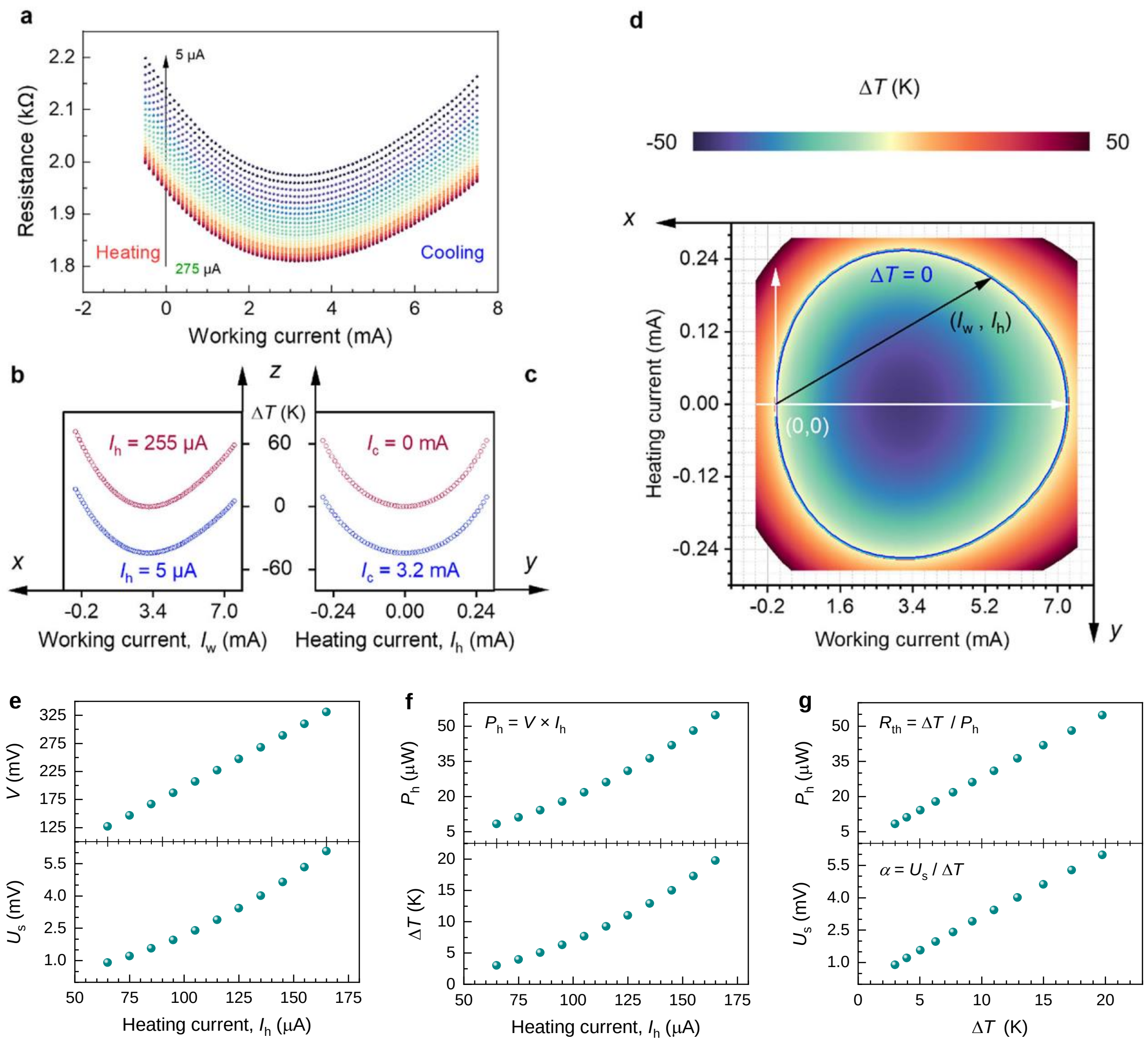

Fig. S6 Heat-compensation method. **a** The real-time resistance value of the Pt temperature sensor using different heating currents (I_h, 5–275 μA, an interval is 10 μA) and working current (I_w) at 380 K, detailed data for Fig. 2a. **b**, **c** The relationship between the temperature control value and two currents in **b** (*xz*-projection of Fig. 2a) and **c** (*yz*-projection of Fig. 2a). **d** Critical conditions for cooling power test under the condition of constant temperature difference. **e** Voltage V of Pt sensor and Seebeck voltage U_s of the μ-TCer as a function of I_h, and the corresponding heating power ($P_h = V \times I_h$) and temperature difference ΔT in **f**. **g** The approximately linear relationship of P_h and U_s with ΔT, respectively, for calculating the intrinsic thermal resistance ($R_{th} = \Delta T / P_h$) and Seebeck coefficient ($\alpha = U_s / \Delta T$) of the μ-TCer, details are provided in Section S1

Fig. S7 Cooling power and *COP*

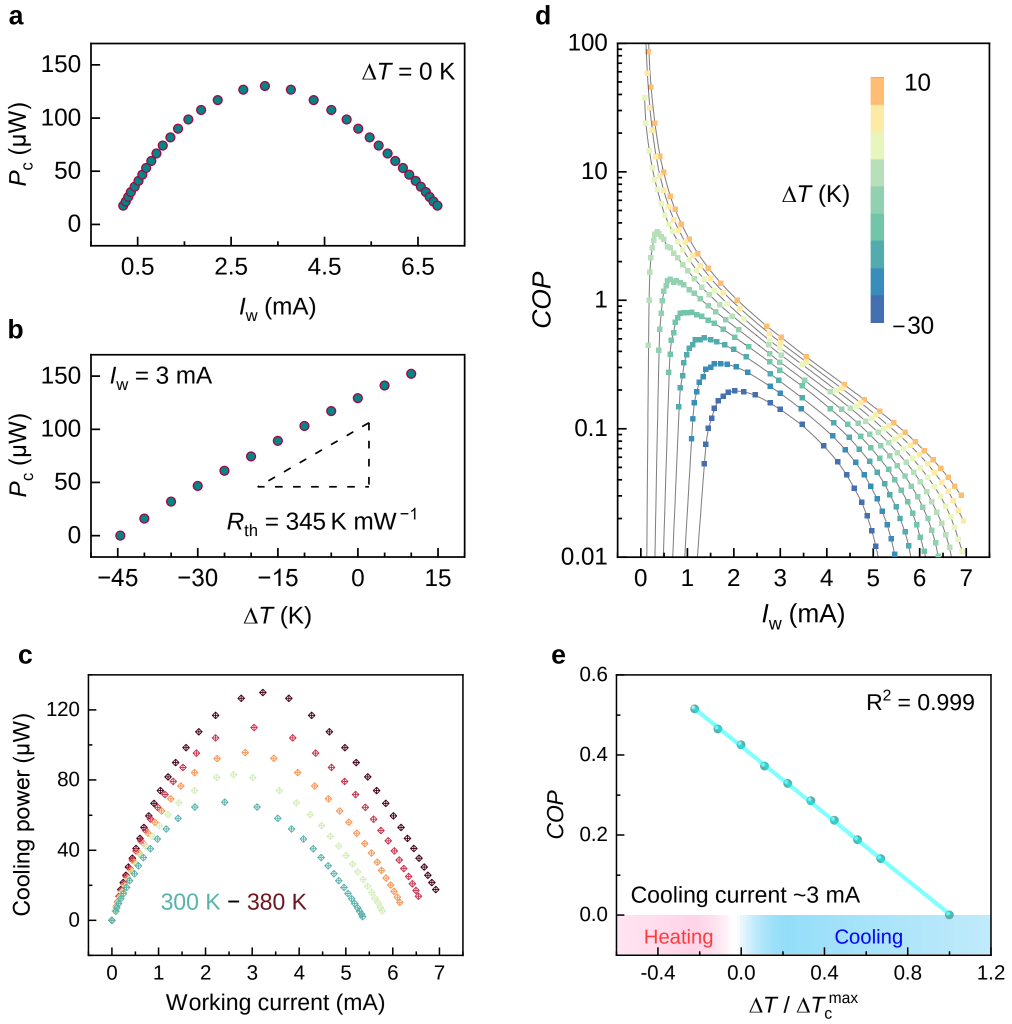

Fig. S7 Cooling power and efficiency test via heat-compensation. **a**, **b** Cooling power P_c as a function of working current I_w ($\Delta T = 0$ K) in **a** and temperature difference ΔT ($I_w = 3$ mA) in **b** at 380 K. Their relationship reflected in the slope is approximately the $1/R_{th}$ value of the μ-TCer. **c** Thermoelectric cooling power when ΔT is 0 K at different ambient temperatures (280–380 K). **d** Coefficient of performance *COP* versus I_w at various ΔTs (−30 to 10 K). The μ-TCer can achieve ultra-high efficiency (*COP* >5) temperature control under small positive ΔTs (≤10 K). It can also achieve a *COP* >1 even under negative ΔTs, which is impossible for passive cooling methods. **e** Linear relationship of *COP* valve and normalized ΔT ($\Delta T / \Delta T_c^{max}$)

Fig. S8 Power and efficiency

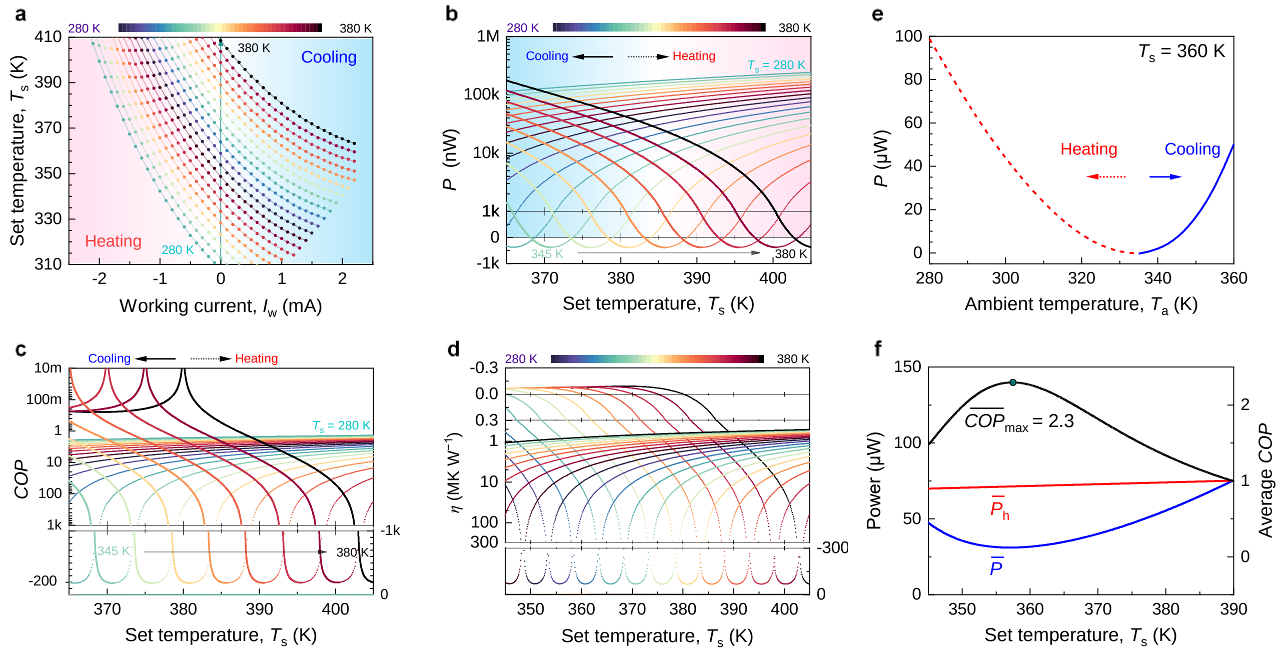

Fig. S8 Power and efficiency of the on-chip micro thermoelectric temperature controller. **a** Working current-dependent controlling temperatures at different ambient temperatures (heating current is 195 μA) and corresponding fitted curves, **b** Setting temperature-dependent electric power consumption *P* at different ambient temperatures, calculated based on data in **a**, and corresponding coefficient of performance *COP* (**c**) and temperature control capability *η* (**d**). **e** Power consumption *P* versus T_a (T_s = 360 K). **f** T_s-dependent average values (T_a from 280 K to 360 K) of power consumption ($\overline{P}$, blue), heating power ($\overline{P_h}$, red) and *COP* ($\overline{COP} = \overline{P_h} / \overline{P}$, black), showing a lowest $\overline{P}$ of ~30 μW when T_s is set to ~357 K. Detail methods are provided in Section S1

Fig. S9 Temperature stability

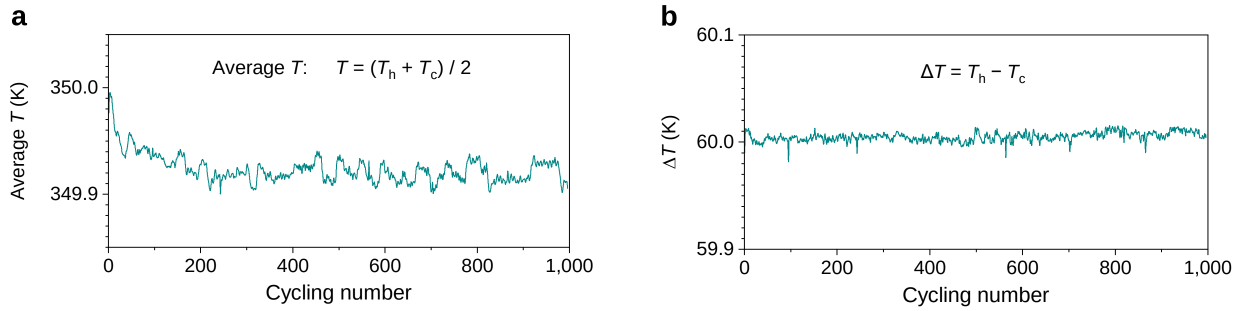

Fig. S9 Temperature stability of the on-chip micro thermoelectric temperature controller. **a** Fluctuating average values and **b** relatively stable difference values based on heating temperature T_h and cooling temperature T_c (Fig. 5a) various cycling numbers

Fig. S10 XRD

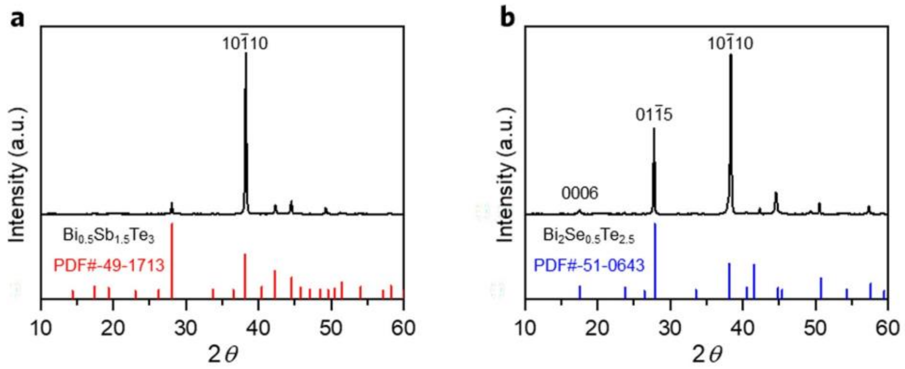

Fig. S10 X-ray diffraction (XRD) patterns of the (**a**) n-type and (**b**) p-type TE films

Fig. S11 EDS

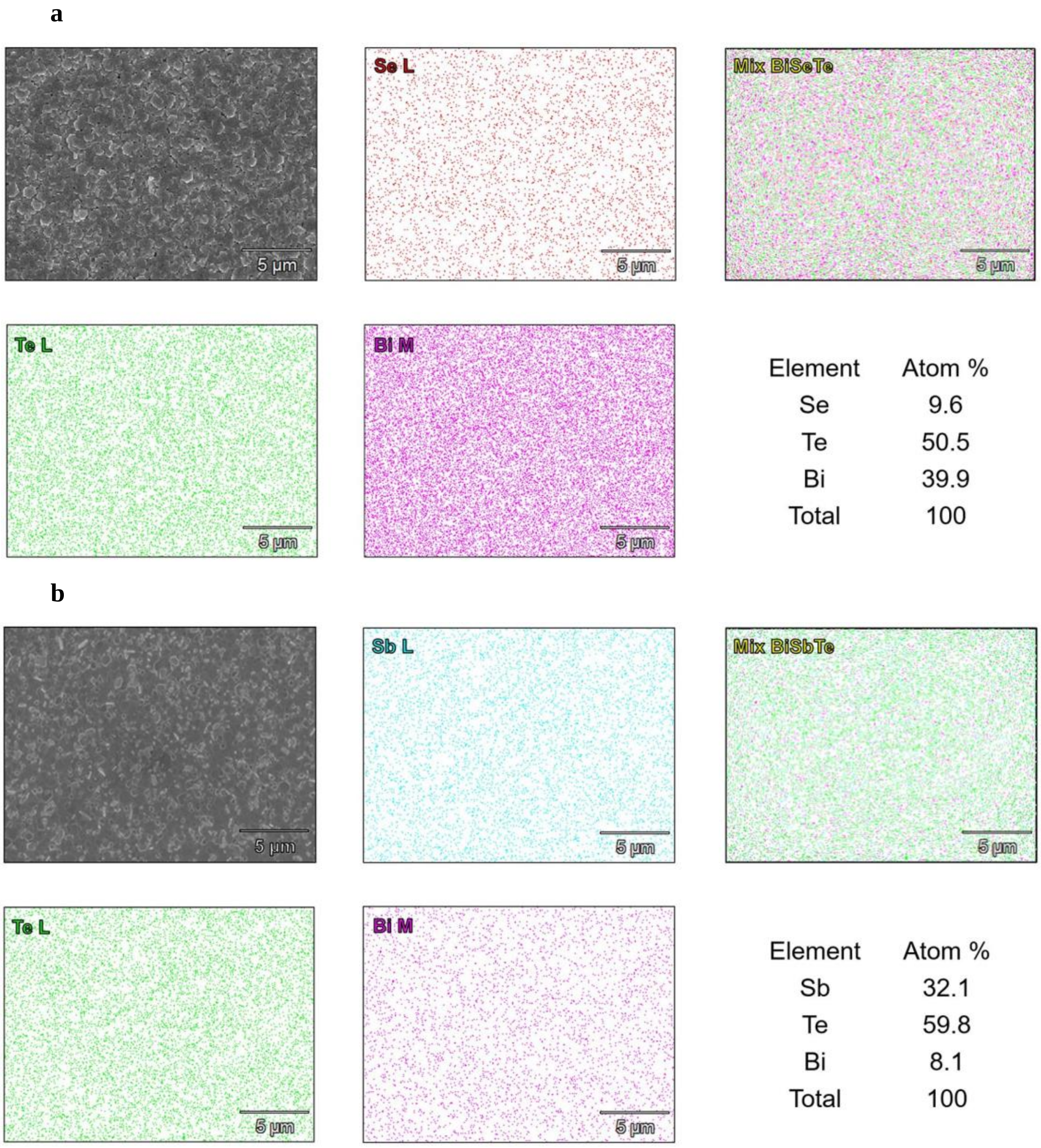

Element	Atom %
Se	9.6
Te	50.5
Bi	39.9
Total	100

Element	Atom %
Sb	32.1
Te	59.8
Bi	8.1
Total	100

Fig. S11 SEM images and EDS mapping results of the (**a**) n-type (Se-doped) and (**b**) p-type (Sb-doped) Bi_2Te_3 TE films, respectively

Fig. S12 Cooling results versus working pressure

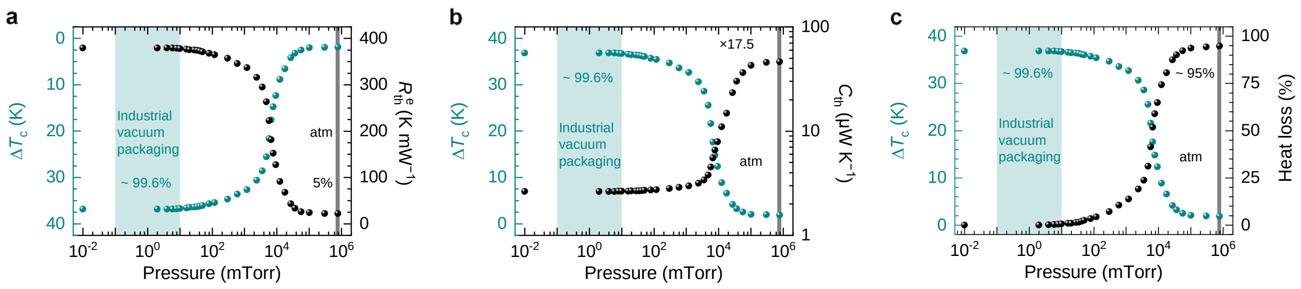

Fig. S12 Cooling results versus working pressure. **a–c** Experimental data of cooling temperature difference ΔT_{c} (green) and equivalent thermal resistance $R_{\mathrm{th}}^{\mathrm{e}}$ (black) in **a** equivalent thermal conductance $C_{\mathrm{th}}^{\mathrm{e}}$ (black) in **b** and heat loss ratio (black, $P_{\mathrm{L}} / P_{\mathrm{c}}$) in **c** due to the heat convection and conduction versus working pressure, details are provided in Section S1

Fig. S13 Comparison of the cooling performance

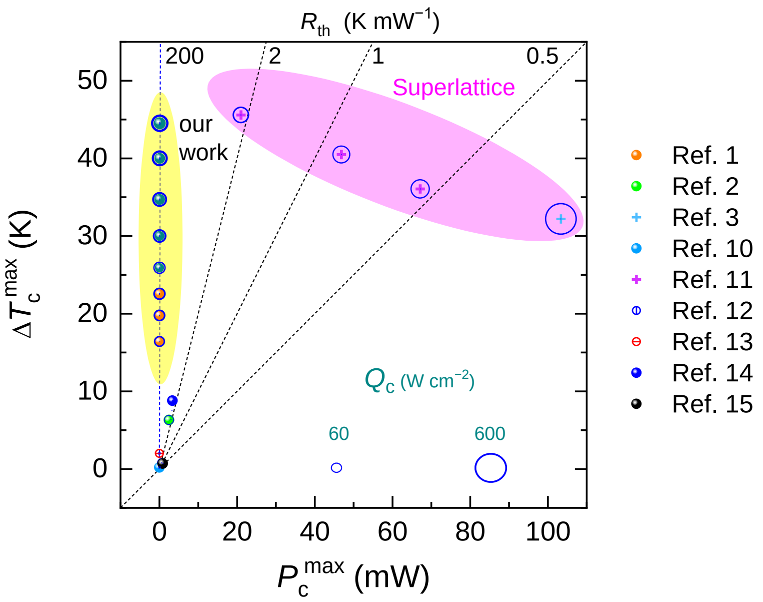

Fig. S13 Comparison of the cooling performance of previously reported micro TE coolers, including the maximum cooling temperature difference $\Delta T_{\mathrm{c}}^{\mathrm{max}}$ (cooling power is zero) and the maximum cooling power $\Delta P_{\mathrm{c}}^{\mathrm{max}}$ (ΔT is zero) of unicouple TEC (one pair of TE legs). The area sizes of the blue bubbles outside the symbols reflect their cooling power density Q_{c} . Corresponding thermal resistances for these one-pair TE legs are also indicated. Details, full references and further performance are provided in Tables S1 and S3

Fig. S14 Original data

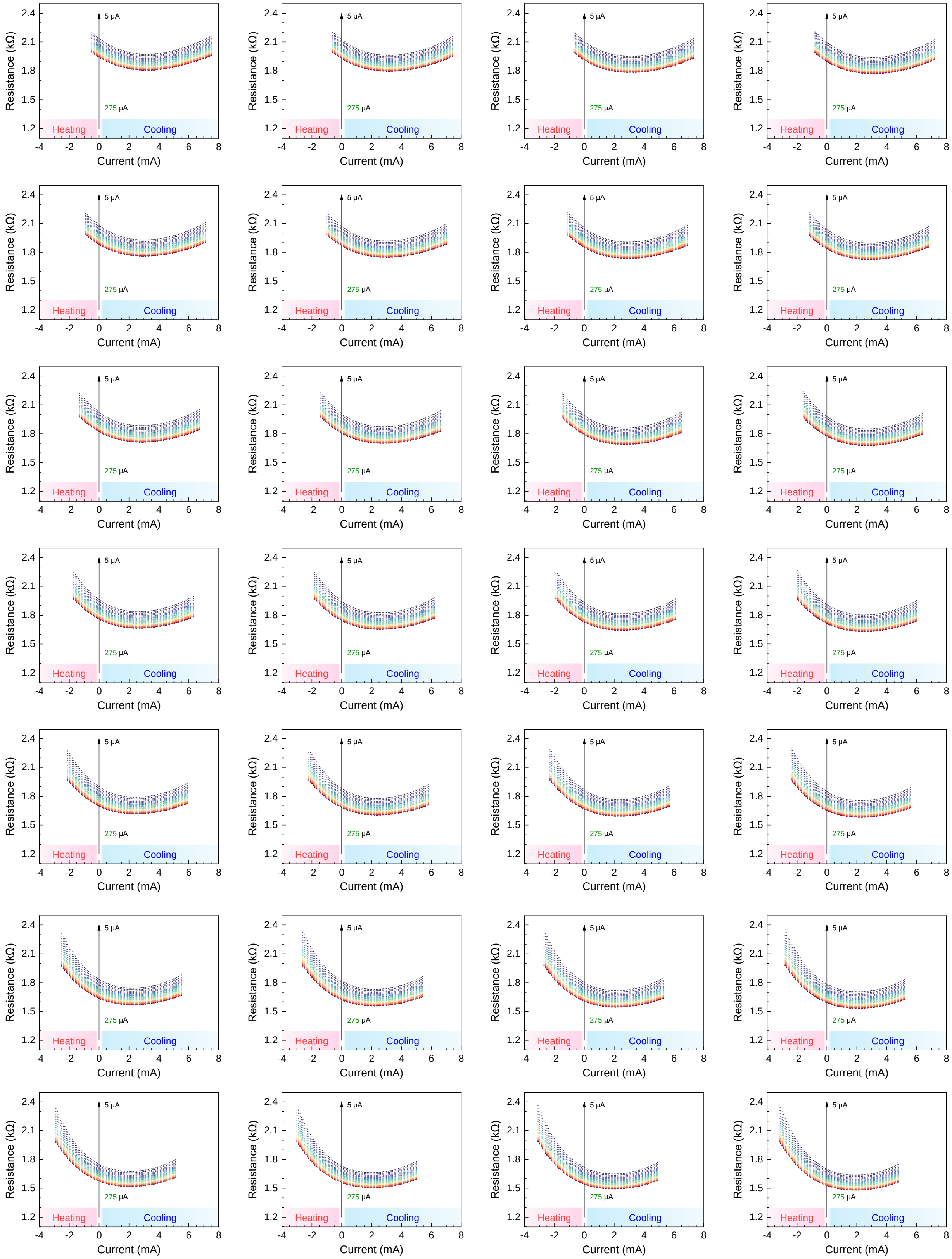

Fig. S14 The original real-time resistance data of the Pt temperature sensor at different ambient temperatures (280-380 K, an interval is 5 K) using different heating currents (5-275 μA, an interval is 10 μA)

Supplementary References

[S1] Q. Jin, Y. Zhao, X. Long, S. Jiang, C. Qian, et al. Flexible carbon nanotube-epitaxially grown nanocrystals for micro-thermoelectric modules. Adv. Mater. **35**, 2304751 (2023). https://doi.org/10.1002/adma.202304751

[S2] G. Li, J. Garcia Fernandez, D.A. Lara Ramos, V. Barati, N. Pérez, et al. Integrated microthermoelectric coolers with rapid response time and high device reliability. Nat. Electron. **1**, 555–561 (2018). https://doi.org/10.1038/s41928-018-0148-3

[S3] R. Venkatasubramanian, E. Siivola, T. Colpitts, B. O'quinn. Thin-film thermoelectric devices with high room-temperature figures of merit. Nature. **413**, 597–602 (2001).

[S4] A.S. Dutt, K. Deng, G. Li, N.B. Pulumati, D.A.L. Ramos, et al. Geometric study of polymer embedded micro thermoelectric cooler with optimized contact resistance. Adv. Electron. Mater. **8**, 2101042 (2022). https://doi.org/10.1002/aelm.202101042

[S5] X. Wang, V. Askarpour, J. Maassen, M. Lundstrom. On the calculation of Lorenz numbers for complex thermoelectric materials. J. Appl. Phys. **123**, 055104 (2018). https://doi.org/10.1063/1.5009939

[S6] L.C. Ortiz, H. Kwon, J. Rodriguez, Y. Chen, G.D. Vukasin, et al. Low-power dual mode MEMS resonators with PPB stability over temperature. J. Microelectromech. Syst. **29**, 190–201 (2020). https://doi.org/10.1109/JMEMS.2020.2970609

[S7] P. Guo, H. Meng, L. Dan, H. Xu, J. Zhao. High vacuum packaging of MEMS devices containing heterogeneous discrete components. Applied Sciences. **11**, 8536 (2021). https://doi.org/10.3390/app11188536

[S8] W. Lee, W. Fon, B.W. Axelrod, M.L. Roukes. High-sensitivity microfluidic calorimeters for biological and chemical applications. Proceedings of the National Academy of Sciences. **106**, 15225-15230 (2009). https://doi.org/10.1073/pnas.0901447106

[S9] R.N. Candler, P. Woo-Tae, L. Huimou, G. Yama, A. Partridge, et al. Single wafer encapsulation of MEMS devices. IEEE Tran. Adv. Packag. **26**, 227–232 (2003). https://doi.org/10.1109/TADVP.2003.818062

[S10] W. Jin, L. Liu, T. Yang, H. Shen, J. Zhu, et al. Exploring Peltier effect in organic thermoelectric films. Nat. Commun. **9**, 3586 (2018). https://doi.org/10.1038/s41467-018-05999-4

[S11] G. Bulman, P. Barletta, J. Lewis, N. Baldasaro, M. Manno, et al. Superlattice-based thin-film thermoelectric modules with high cooling fluxes. Nat. Commun. **7**, 10302 (2016). https://doi.org/10.1038/ncomms10302

[S12] W. Hou, X. Nie, W. Zhao, H. Zhou, X. Mu, et al. Fabrication and excellent performances of $Bi_{0.5}Sb_{1.5}Te_3$/epoxy flexible thermoelectric cooling devices. Nano Energy. **50**, 766-776 (2018). https://doi.org/10.1016/j.nanoen.2018.06.020

[S13] G.J. Snyder, J.R. Lim, C.K. Huang, J.P. Fleurial. Thermoelectric microdevice fabricated by a MEMS-like electrochemical process. Nat. Mater. **2**, 528–531 (2003). https://doi.org/10.1038/nmat943

[S14] R.A. Kishore, A. Nozariasbmarz, B. Poudel, M. Sanghadasa, S. Priya. Ultra-high performance wearable thermoelectric coolers with less materials. Nat. Commun. **10**, 1765 (2019). https://doi.org/10.1038/s41467-019-09707-8

[S15] Y. Chen, X. Nie, C. Sun, S. Ke, W. Xu, et al. Realizing high-performance BiSbTe magnetic flexible films via acceleration movement and hopping migration of carriers. Adv. Funct. Mater. **32**, 2111373 (2022). https://doi.org/10.1002/adfm.202111373
